# Supplementary material for: Physical activity, diet and BMI in children aged 6–8 years: a cross-sectional analysis
Source: BMJ Open. 2014 Jun 5;4(6):e005001. doi: 10.1136/bmjopen-2014-005001 (PMC4054632; doi:10.1136/bmjopen-2014-005001)
Supplement: Author's manuscript [file bmjopen-2014-005001.draft_revisions.pdf]

Impact of physical activity and diet on BMI in 6-8 year old children; a cross-sectional analysis

Journal:	*BMJ Open*
Manuscript ID:	bmjopen-2014-005001
Article Type:	Research
Date Submitted by the Author:	08-Feb-2014

Complete List of Authors:	Basterfield, Laura; Newcastle University, Institute of Health & Society<br>Jones, Angela; Newcastle University, Institute of Health & Society<br>Parkinson, Kathryn<br>Pearce, Mark; Newcastle University, Institute of Health & Society<br>Reilly, John; University of Strathclyde,<br>Adamson, Ashley; Newcastle University, Institute of Health & Society

**Primary Subject Heading**:	Paediatrics
Secondary Subject Heading:	Public health
Keywords:	Community child health < PAEDIATRICS, PUBLIC HEALTH, EPIDEMIOLOGY

SCHOLARONE™
Manuscripts

For peer review only - http://bmjopen.bmj.com/site/about/guidelines.xhtml

Impact of physical activity and diet on BMI in 6-8 year old children; a cross-sectional analysis

Corresponding author:
Ashley J Adamson, Institute of Health & Society, Human Nutrition Research Centre, Newcastle University, Newcastle upon Tyne, UK NE2 4HH.
Ashley.Adamson@ncl.ac.uk, Tel: 0191 208 5276, Fax: 0191 208 6043.

Co-authors:
Laura Basterfield, Institute of Health & Society, Human Nutrition Research Centre, Newcastle University, Newcastle upon Tyne, UK
Angela R Jones, Institute of Health & Society, Human Nutrition Research Centre, Newcastle University, Newcastle upon Tyne, UK
Kathryn N Parkinson, Institute of Health & Society, Human Nutrition Research Centre, Newcastle University, Newcastle upon Tyne, UK
Mark S Pearce, Institute of Health & Society, Newcastle University, Sir James Spence Institute, Royal Victoria Infirmary, Newcastle upon Tyne, UK
John J Reilly, Physical Activity for Health Group, School of Psychological Sciences and Health, University of Strathclyde, Glasgow, UK
The Gateshead Millennium Study Core Team: Ashley Adamson, Anne Dale, Robert Drewett, Ann Le Couteur, Paul McArdle, Kathryn Parkinson, Mark Pearce, John Reilly, Charlotte Wright

Key words: epidemiologic studies; child; overweight; physical activity; diet

Word count: 3702

For peer review only

ABSTRACT

Objective

To assess the influence of current PA and dietary intake on body mass index (BMI) in English children.

Design and setting

Longitudinal birth cohort study in northeast England, cross-sectional analysis.

Participants

425 children (41% of the original cohort) aged 6-8 years (49% boys).

Main outcome measures

PA over seven days was measured objectively by accelerometer, three categories of PA were created: 'Active' ≥60min/d moderate-to-vigorous-intensity PA (MVPA); 'moderately active' 30-59min/d MVPA; inactive <30min/d MVPA. Dietary intake over four days was measured using a prospective dietary assessment tool which incorporated elements of both the food diary and food frequency methods. Three diet categories were created: 'healthy'; 'unhealthy' and 'mixed', according to the number of portions of different foods consumed. Adherence to the '5 a day' recommendations for portions of fruit and vegetables was also assessed. Children were classified as 'healthy weight' or 'overweight or obese' (OW/OB) according to International Obesity Taskforce cutpoints for BMI. Associations between weight status and PA/diet categories were analysed using logistic regression.

Results

Few children met the UK recommended guidelines for either MVPA or fruit and vegetable intake, with just 7% meeting the recommended amount of MVPA of 60 mins/d, and 3% meeting the 5-a-day fruit and vegetable recommendation. Higher PA was associated with a lower odds ratio for OW/OB in boys only (0.20 95% CI 0.04-0.88). There was no association detected between dietary intake and OW/OB in either sex.

Conclusions

For peer review only

Increasing MVPA may help to reduce OW/OB in boys; however, more research is required to examine this relationship in girls. Children are not meeting the UK guidelines for diet and PA, and more needs to be done to improve this situation.

Strengths and limitations of this study

- Direct objectively measured physical activity and observed dietary intake
- Direct measures of body composition
- Population based birth cohort socioeconomically representative of northeast England
- Ethnically homogeneous sample which reduces generalisability
- Cross-sectional so cannot determine direction of causality

For peer review only

INTRODUCTION

Childhood obesity is a major public health challenge both in the UK and around the world, with governments increasingly involving themselves in different strategies to reduce excess weight in the population.[1, 2] Recent findings from the National Child Measurement Programme (2011-2012) regarding the prevalence of childhood overweight and obesity are concerning,[3] especially in northeast England, which is the worst-affected region of England outside of London, where 24.5% of 4-5 year olds and 37.0% of 10-11 year olds are overweight or obese. There remains a limited understanding of the timing and causes of childhood obesity and there is an urgent need for effective preventive strategies.

Current UK health recommendations state that children should accumulate at least 60 minutes of moderate-vigorous intensity physical activity (MVPA) per day, with vigorous intensity activities performed on at least 3 days per week.[4] This recommendation is based on evidence from intervention studies.[5] However, recent evidence suggests that the majority of children fail to meet this target, with 51% of English 4-10 year old boys and 34% of 4-10year old girls achieving the physical activity recommendations as measured by accelerometer, figures which dropped to 7% and 0% among 11-15 year olds.[6] In addition, children should consume a healthy balanced diet which contains a variety of foods, including at least 5 portions of fruit and vegetables per day, plenty of starchy foods (particularly wholegrain varieties), some protein-rich foods and some milk and dairy foods. It is also recommended that foods high in fat (especially saturated fat), salt and sugar should only be consumed in small amounts.[7] Data from the National Diet and Nutrition Survey in 2010 indicated that children aged 4-10 years consume an average of 173g of fruit and vegetables daily, equivalent to 2.2 80g portions (or 2.8 including fruit juice).[8] Data from longitudinal cohorts are beginning to uncover the associations between low levels of physical activity and unfavourable body composition e.g. Janz et al (2009),[9] but the data on the relationship between diet/dietary components and body composition have so far been equivocal, as reviewed by Must et al.[10] This may be due to the contrasting methods of dietary assessment and analysis and the complexity of dietary patterns; given the importance of energy balance in maintenance of a healthy body weight it is vital that measurement of dietary intake continues to be improved. Evidence of the impact of *both* objectively measured physical activity (PA)/sedentary behaviour (SB) *and* food intake on body composition in primary school-age children is lacking, with most studies measuring either physical

activity *or* food intake. A recent Scottish study assessed the effect of dietary patterns and physical activity on body mass index (BMI),[11] but used a questionnaire that is known to overestimate time spent in physical activity.[12] The authors did not find a consistent association between dietary patterns or time spent in physical activity and BMI group.[11]

The primary aim of the present study was to describe the relationship between overweight and obesity, BMI, PA, and dietary habits in a sample of children that are socioeconomically representative of northeast England.[13]

METHODS

Participants

The Gateshead Millennium Study (GMS) cohort originally consisted of 1029 babies and their families and is described in detail elsewhere.[13] Briefly, all mothers resident in Gateshead who gave birth within pre-specified recruiting weeks between June 1999 and May 2000 were invited to take part, 81% (n=1011) agreed. Participants were mostly from the white ethnic majority (98%). Data collection for the present study involved a variety of lifestyle and anthropometric measures, and took place between October 2006 and December 2007 when the children were aged 6-8 years. The data presented here are cross-sectional data from this data collection phase only. Ethical approval was given by Gateshead and South Tyneside Local National Health Service Research Ethics Committee. Parents provided informed written consent for their child’s participation and children gave their written assent.

Measures

Physical activity and sedentary behaviour

PA and SB were objectively measured using Actigraph GT1M accelerometers (Actigraph LLC, Pensacola, Florida, USA) attached to an elastic belt and worn on the right hip as described previously.[14] Children were asked to wear the accelerometer during waking hours for 7 days and to remove them only for water-based activities. Parents were asked to complete a time-log detailing when the accelerometer had been worn.

For peer review only

The accelerometers collected data in 15 second sampling intervals (epochs); prior to analysis the data were collapsed to 60 second epochs. Data were verified manually using the time-log and accelerometry output as described previously[12] to delete occasional periods of non-wear time.

Three constructs of PA and one of SB were generated: total volume of activity (expressed as mean daily accelerometer counts per minute, cpm); MVPA daily, equivalent to energy expenditure in excess of approximately three times resting energy expenditure (expressed as both minutes per day and % of daily time spent in MVPA), and sedentary behaviour (equivalent to no translocation of the trunk[15] (expressed as % daily time spent in SB)).

The cutpoint of ≥3200 cpm[16] was used to define MVPA; and <1100 cpm[17] was used to define SB. The Actigraph GT1M model has been shown to have a consistent 9% bias relative to model 7164[18] from which the cutpoints were derived, so a +9% correction was applied to the raw accelerometry data before applying cutpoints. Of 606 accelerometers given out, 510 were worn for at least 3 days and for at least 6 hours per day[19] and thus were deemed eligible for analysis in the present study.

Dietary intake

Food intake in the children was assessed over 4 days (2 week days and 2 weekend days) using the ‘Food Assessment in Schools Tool’ (FAST) food diary.[20] The FAST food diary is a prospective dietary assessment method which incorporates elements of both the food diary and the food frequency methods and was specifically designed to assess the diets of primary school children. Briefly, each day is split into six timeslots, each containing a simple tick list of up to 50 foods commonly eaten by children of this age.[21] Space for additional foods was also provided. Researchers and lay-observers recorded food intake for each child during the school day, and parents and other carers recorded food intake at all other times. No interviews or estimations of portion size were required during data collection, average age- and sex- specific portion sizes for all foods consumed were ascertained using relevant UK National Diet and Nutrition Survey data.[20, 21] This method allowed analysis of food choice at individual level but not of energy intake.

A ‘healthy’ and ‘unhealthy’ dietary indicator score was created for each of the four days, and a daily mean calculated. The ‘healthy’ indicator (HI) score was calculated by summing the number of portions consumed of the following foods: wholemeal bread; non-high sugar wholegrain breakfast cereals; fruit; vegetables; semi-skimmed

milk as a drink; semi-skimmed milk; non-processed meat and fish products; beans and pulses; water and white bread with added fibre. The 'unhealthy' indicator (UI) score was calculated by summing the number of portions consumed of the following foods: white bread; high-sugar refined breakfast cereals; fried chips; biscuits; confectionery, cakes and sweet puddings; full-fat milk as a drink; full-fat milk; processed meat and fish products; full sugar cordial or squash; full sugar carbonated drinks; chocolate/milkshake powder; crisps and savoury snacks and sugar. To assess adherence to dietary guidelines, fruit and vegetable consumption was measured in 80g amounts, fruit juice in 150ml portions. Only one portion of fruit juice may count towards daily fruit and vegetable intake.[7]

Anthropometry

All body measurements were taken in duplicate with the children in light indoor clothing, without socks and shoes. Height was measured to 0.1cm with a Leicester portable height measure (Chasmors, London, UK), waist to 0.1cm at the minimum circumference between the lowest rib and iliac crest, and weight to 0.1kg using a TANITA TBF 300MA body fat analyser (Chasmors, London, UK). BMI was calculated (wt (kg)/ht(m^2)). Categories of underweight, healthy weight, overweight and obesity were calculated according to the International Obesity Taskforce (IOTF) criteria.[22, 23]

Statistical analysis

The sample size for the current cross-sectional study was fixed by the size of the cohort, participant attrition and those children providing complete data for PA, dietary intake and anthropometry at this data sweep.

Analysis was performed using SPSS 17 and STATA 10. As data were skewed, Mann-Whitney tests were performed to test for differences between sexes and Wilcoxon Rank Tests to assess differences in weekend and weekday PA/SB. Median and interquartile ranges are reported unless otherwise stated.

Logistic regression was used to predict the likelihood of being overweight or obese (OW/OB) at different levels of PA and with different dietary indicator scores. Children were assigned to one of three PA categories: (1) 'active': those who met the ≥60 minutes per day guidelines (this figure was generated by dividing the total amount of MVPA for the week by the number of days the accelerometer was worn.); (2) 'moderately active': those who did between 30 and 60 min·d^{-1}; (3) 'inactive': those who did less than 30 min·d^{-1}. Children were also assigned to one of three dietary categories: (1) 'healthy': those children whose HI score was ≥ the median and whose

For peer review only

UI score was < the median ; (2) 'unhealthy': those children whose UI score was ≥ the median and whose HI score < the median ; (3) 'mixed': those diets which neither met the criteria for a 'healthy' diet or 'unhealthy' diet. Children were lastly classified by BMI as: (1) not overweight or obese; (2) overweight or obese. Odds ratios (OR) and corresponding 95% confidence intervals (95% CI) are reported. Significance was set at $p<0.05$. Sex was included in the regression analysis as a potential confounder. Townsend score from birth (a measure of deprivation) was unrelated to any of the variables and thus not included in the final analysis.

RESULTS

Overall, 510 children completed sufficient accelerometry recording, 474 children returned completed FAST food diaries and 597 children had anthropometry measurements. Data from all three domains were provided by 425 children and were used in the analysis. There were no significant differences in food intake, PA or BMI between children providing complete records and those who did not. Children had a mean age of 7.4y (range 6.4-8.4). Characteristics of the sample are displayed in Table 1.

For peer review only

Table 1 Participant characteristics (median and IQR), by sex

	Combined* (n=425)		Boys (n=211) (49.6%)		Girls (n=214) (50.4%)		p-value
Height (cm)	1.24	1.21, 1.29	1.26	1.22, 1.29	1.24	1.21, 1.28	0.039
Weight (kg)	26.4	22.7, 28.7	25.7	23.0, 29.1	25.0	22.5, 28.4	0.264
Waist (cm)	55.7	52.6, 59.0	56.5	53.5, 59.4	54.8	52.1, 58.7	0.006
BMI (kg/m^2)	16.2	15.3, 17.8	16.1	15.4, 17.8	16.3	15.2, 17.8	0.878

*n for individual measures varies from 421-425. Mann-Whitney tests performed. BMI, body mass index

For peer review only

For peer review only - http://bmjopen.bmj.com/site/about/guidelines.xhtml

Using IOTF cutoffs,[22] 24% of the children were overweight or obese, with no significant difference between girls and boys (Table 2).

Table 2 Distribution of children between weight categories, by sex

	Combined		Boys (49.6%)		Girls (50.4%)	
	n	%	n	%	n	%
Underweight	3	0.7	1	0.5	2	0.9
Healthy weight	320	75.3	160	75.8	160	74.8
Overweight	75	17.6	35	16.6	40	18.7
Obese	27	6.4	15	7.1	12	5.6
Total	425	100	211	100	214	100

There were no significant differences between the sexes in the number of days the activity belt was worn or the total volume of activity (mean cpm) (Table 3).

For peer review only

Table 3 Comparison of physical activity, sedentary behaviour and dietary intake between sexes (median and IQR)

	Combined (n=425)		Boys (n=211)		Girls (n=214)		p-value
Number of days accelerometer worn	7	6,7	7	6,7	7	6,7	0.380
Hours worn/d	11.3	10.6, 11.9	11.4	10.7, 12.0	11.1	10.4, 11.8	0.077
Mean cpm/d	727	604, 878	740	624, 881	718	591, 868	0.313
MVPA (min/d)	26	18, 38	28	19, 43	25	16, 36	0.020
% time in MVPA	3.9	2.6, 5.7	4.2	2.7, 6.3	3.8	2.5, 5.5	0.043
% time SB	77.7	73.6, 81.8	76.9	71.9, 80.8	78.4	74.6, 82.7	0.002
Achieved 60 min/d MVPA*	31	(7.3%)	23	(10.9%)	8	(3.7%)	0.005
Portions of fruit & vegetables consumed/d	1.7	1.1, 2.5	1.7	1.0, 2.5	1.8	1.2, 2.5	0.211
Portions of fruit & vegetables consumed/d$	2.1	1.4,3.0	2.0	1.3,2.9	2.2	1.4,3.1	0.224
Ate 5 portions of fruit &vegetables/d$	13	(3.1%)	8	(3.8%)	5	(2.3%)	0.384
Number of healthy indicator foods/d	5.0	3.8, 6.5	5.0	3.8, 6.5	5.1	3.8, 6.5	0.342
Number of unhealthy indicator foods/d	5.3	4.3, 6.3	5.5	4.3, 6.3	5.3	4.3, 6.3	0.496

MVPA, moderate-vigorous intensity physical activity

*total MVPA for the week/days worn, $including one 150ml portion of juice. Mann-Whitney tests performed.

For peer review only - http://bmjopen.bmj.com/site/about/guidelines.xhtml

Levels of activity in general were low, as reported previously; [12] only 31 children (7%) achieved an average of at least 60 min MVPA·d^{-1}, 23 boys and 8 girls. Only two children (0.5%) accumulated at least 60 minutes on every day they wore the accelerometer. The majority of time was spent in sedentary behaviour (median 78% of wear time, equivalent to around 8.6 hours per day). When looking at the differences between weekend and weekday activity, volume of activity (cpm) was similar, and slightly but significantly more MVPA was taken on weekdays ($p<0.0001$) (data not shown). Girls had a greater volume of activity at the weekend but a lower time spent in MVPA, suggesting the majority of their activities are in the sedentary or light intensity categories. Boys spent significantly more time in MVPA on weekdays than girls.

Dietary intake was similar for boys and girls (Table 3). Children consumed a median of 1.7 portions of fruit and vegetables daily (or 2.1 portions when including fruit juice), with just 13/425 children (3.1%) consuming their ‘5 a day’. On average, children ate 5.0 healthy indicator foods and 5.3 unhealthy indicator foods daily.

Table 4 displays the distribution of overweight/obesity by diet and activity group.

For peer review only

Table 4. Distribution of OW/OB by physical activity and dietary category (%)

	Healthy weight				Overweight or obese			
	Dietary Pattern				Dietary Pattern			
PA category	Unhealthy	Mixed	Healthy	Total	Unhealthy	Mixed	Healthy	Total
**Boys**								
Inactive	16.8	20.5	11.2	48.4	22.0	26.0	28.0	76.0
Moderately active	10.6	17.4	10.6	38.5	4.0	14.0	2.0	20.0
Active	5.6	6.2	1.2	13.0	0.0	2.0	2.0	4.0
Total	32.9	44.1	23.0	100.0	26.0	42.0	32.0	100.0
**Girls**								
Inactive	16.7	17.9	24.7	59.3	17.3	34.6	19.2	71.2
Moderately active	10.5	19.1	7.4	37.0	7.7	7.7	9.6	25.0
Active	0.6	1.9	1.2	3.7	0.0	3.8	0.0	3.8
Total	27.8	38.9	33.3	100.0	25.0	46.2	28.8	100.0

For peer review only

For peer review only - http://bmjopen.bmj.com/site/about/guidelines.xhtml

13% of healthy weight boys were in the ‘active’ PA category, compared with just 4% of OW/OB boys. 48% of healthy weight boys were inactive compared with 76% of OW/OB boys. There was less variation for girls, as only 8 girls in total were in the active category. 59% of healthy weight girls were in the inactive category compared with 71% of OW/OB girls.

Compared with the reference group (inactive), children in the moderately active group had a significantly lower risk of being OW/OB (OR=0.44, 0.26-0.74 (Table 5)).

For peer review only

Table 5 Likelihood of being overweight or obese for different activity levels and dietary patterns (odds ratios and 95% CI)

	Combined (n=425)	p-value	Boys (n=211)	p-value	Girls (n=214)	p-value
Inactive (reference)	1		1		1	
Moderately active	0.44 (0.26-0.74)	0.002	0.33 (0.15-0.72)	0.005	0.56 (0.28-1.14)	0.112
Active	0.34 (0.12-1.02)	0.054	0.20 (0.04-0.88)	0.033	0.86 (0.17-4.48)	0.863
Unhealthy diet (reference)	1		1		1	
Mixed diet	1.27 (0.73-2.19)	0.40	1.21 (0.55-2.62)	0.637	1.32 (0.61-2.86)	0.485
Healthy diet	1.28 (0.71-2.33)	0.41	1.76 (0.76-4.10)	0.188	0.96 (0.42-2.23)	0.927

For peer review only

For peer review only - http://bmjopen.bmj.com/site/about/guidelines.xhtml

Results for children in the active group (≥60 min MVPA·d^{-1} (OR=0.34, 95% CI 0.12-1.02) approached significance (p=0.054). Similar results were seen after adjusting for sex; PA OR 0.96 p=0.876, diet OR 1.02 p=0.921.

When analysing girls and boys separately, the association between PA and risk of OW/OB remained only for boys. Boys who were moderately active had an OR of 0.33 (0.15-0.72) for risk of OW/OB (p=0.005), and boys in the active category had an OR of 0.20 (0.04-0.88) (p=0.033), compared with the inactive group. Moderately active girls had an OR of 0.56 (0.28-1.14) (p=0.112) and active girls an OR of 0.86 (0.17-4.48) (p=0.863) for OW/OB compared with inactive girls.

There was no influence of dietary pattern on risk of OW/OB: OR 1.27 (0.73-2.19 p=0.40) for the mixed diet group, 1.28 (0.71-2.33) for the healthy group (p=0.41) compared with the reference group (unhealthy diet) (Table 5), with similar results after adjusting for sex.

There was no evidence of an interaction between PA and food intake on risk of OW/OB.

DISCUSSION

Our results provide evidence that children in the north east of England are not meeting the guidelines for physical activity and nutrition that are recommended for good health. Lack of moderate-vigorous intensity physical activity was associated with an increased risk of being overweight and obese in boys. Lack of effect in girls could be due to the low numbers being sufficiently active.

The present study has limitations in terms of the ethnically homogenous sample, which may limit the generalisability of the findings to other samples and settings. There was also a lack of variability in PA, with data skewed towards inactivity, particularly in girls. There are several cut-points in use for classifying MVPA[24] and the value of 3200cpm used in the current study was obtained through comparison with energy expenditure,[16] and similar to those from other studies.[15] Given the on-going discussion regarding the choice of cut-point there may be no suitable cut-point for all ages of childhood. As the data are cross-sectional, it is not possible to determine the direction of causality, which will be addressed by future follow up. However, the strengths of this study include the relatively large sample size, the focused age of the children, and the use of objective measures of PA (accelerometry) combined with dietary observations is unique.

For peer review only

Few other studies have used objective methods to describe levels of habitual physical activity and sedentary behaviour in 6-8 year olds in combination with dietary intake measures. Recent UK studies in 3-5 year olds[14] and 11-12 year olds[25] which have used the same objective methods of PA measurement have found similarly low levels of physical activity with adherence to the 60 minutes/day recommendation of MVPA being achieved in <5% of their samples. In contrast, UK studies which have used subjective methods (questionnaires) have generally reported much higher levels of MVPA, for example the Health Survey for England, which leads to over-estimates of PA.[12] Low levels of physical activity were present in our sample well before puberty, the period traditionally regarded as being associated with low levels of physical activity. In keeping with other studies[9], [26], we found that boys were more active than girls, and the lack of association of PA on risk of OW/OB in the girls compared with the boys suggests further work is required into the mechanisms by which increasing PA and/or decreasing SB reduces risk of OW/OB in children. Few studies have looked at the relationship between the '60min' guidelines and body composition. Martinez-Gomez et al (2010)[27] reported that 60 $min \cdot d^{-1}$ MVPA could discriminate between normal weight and OW/OB adolescents (mean age 14.7 years), however they used a lower cut-point for MVPA of 2000cpm, compared with the 3200cpm used in the present study. This resulted in average $MVPA \cdot d^{-1}$ of 66 minutes for boys and 50 minutes for girls.[27] Recent evidence using 3200cpm as the MVPA cutpoint from 10 year olds in France[28] found that only 10% of the 'normal weight' children reached 60 $min \cdot d^{-1}$, but 44% did at least 30 $min \cdot d^{-1}$. In addition, although only 5% of OW/OB children did at least 60$min \cdot d^{-1}$ MVPA, 34% reached 30 $min \cdot d^{-1}$.[28] Our results show that this lower recommendation could still have benefits in terms of body composition, particularly for boys.

Whilst it is generally accepted that physical activity decreases with age,[29] pinpointing the age when the decrease starts is important for targeted interventions. Our results suggest that even at age 7 years physical activity levels are far lower than recommended. The great proportion of time spent sedentary by both sexes is concerning - in both sexes almost 7 hours per day were spent with no translocation of the trunk.[15] Both sexes took part in more MVPA on weekdays than weekends, highlighting both the importance the school environment plays in maintaining activity levels, and that more needs to be done to increase activity at the weekend. The cross-sectional nature of the present study means we cannot say whether the children have already started to reduce their activity. However, data we have published from the cohort at a 2 year follow-up show a decrease in MVPA from age 7

For peer review only - http://bmjopen.bmj.com/site/about/guidelines.xhtml

to 9 years, and the decrease in MVPA associated with an increase in BMI-z score in boys.[30, 31]

The low consumption of fruit and vegetables, and the relatively high consumption of an unhealthy diet in this sample are both cause for concern, despite the lack of association between the ‘healthy’ or ‘unhealthy’ categories and risk of OW/OB. The lack of association between dietary variables and risk of OW/OB in this study, as in others, may be due to the lack of individual portion sizes which prohibited the assessment of energy intake. The dietary assessment tool used in this study allowed direct observation of intake but applied standard portion sizes from a previous National Diet and Nutrition Survey which used a weighed intake method.[21] The amount for a ‘portion’ of fruits and vegetables was set at 80g, which is considered suitable for children aged over 11 years.[32] A lower amount may be more suitable for children under 11 years, but as this has not yet been identified,[32] 80g was used in this analysis. It is possible that overweight children are eating larger portions of healthy foods, or the parents are aware of their child’s overweight and have reduced food intake accordingly. There may also be an element of mis-reporting or optimistic bias if the parent believes their child is overweight and eats either to excess or ‘unhealthily’. Foods in the current study were assigned to the ‘healthy’ or ‘unhealthy’ group though a combination of current national guidelines for consumption and restriction[7] according to nutritional content. Since these analyses were performed, Frémeaux and colleagues[33] have published a longitudinal factor analysis of English children’s food intake from age 5 to 13 years. They reported the emergence of two main diet types that matched almost identically with ours.[33] Recent evidence from Scottish 5-11 year olds did not find an association between dietary patterns and BMI or physical activity,[11] and observed an inverse association between ‘screen time’ and healthier eating patterns (diet measure with food frequency questionnaire, FFQ, and physical activity measured by questionnaire, PAQ). The ALSPAC cohort in the UK were assessed for associations between PA and diet at age 10-11 years, and found only weak relationships between the two[34] (3x1-day food diary at 10y, accelerometry at 11y). A Greek case-control study found a protective effect of PA[35] on risk of OW/OB, and an increased risk with increased time spent watching TV, and consumption of sugar-sweetened beverages (FFQ/PAQ), an observation also reported in Spanish children (FFQ/PAQ).[36] Others [37] found an effect of sedentary behaviour on risk of OW/OB, but again no effect of diet composition (questionnaire). A varied food pattern plus a physically active lifestyle was inversely related to OW in French children (7-day food diary/PAQ).[38] Similarly, reduced physical activity, but

For peer review only - http://bmjopen.bmj.com/site/about/guidelines.xhtml

not excessive energy intake, was associated with increased risk of OB in Turkish children (3-day food diary/PAQ).[39] However, these studies included a wide age range of children, and physical activity and sedentary behaviour were measured objectively only in the ALSPAC cohort[34], so our observations strengthen the evidence base for the protective effects of PA on risk of OW/OB.

In conclusion, children in northeast England have extremely low levels of habitual MVPA, high levels of sedentary behaviour and consume a diet low in fruits and vegetables but high in foods associated with an unhealthy diet. Increased PA was associated with reduced risk of OW/OB in boys. Further work is required to uncover the associations of PA/diet and OW/OB in girls and the longitudinal associations between PA/diet and OW/OB in boys and girls. These observations give cause for concern in relation to their current and future risk of cardiovascular diseases, diabetes, and obesity. Our observations suggest that UK government targets in relation to levels of physical activity and nutrition among children are not being met.

ACKNOWLEDGEMENTS

The Gateshead Millennium Study is supported by a grant from the National Prevention Research Initiative (incorporating funding from British Heart Foundation; Cancer Research UK; Department of Health; Diabetes UK; Economic and Social Research Council; Food Standards Agency; Medical Research Council; Research and Development Office for the Northern Ireland Health and Social Services; Chief Scientist Office, Scottish Government Health Directorates; Welsh Assembly Government and World Cancer Research Fund). The cohort was first established with funding from the Henry Smith Charity and Sport Aiding Research in Kids (SPARKS) and followed up with grants from Gateshead NHS Trust R&D, Northern and Yorkshire NHS R&D, and Northumberland, Tyne and Wear NHS Trust. We acknowledge the support of an External Reference Group in conducting the study. We appreciate the support of Gateshead Health NHS Foundation Trust, Gateshead Education Authority and local schools. We warmly thank the research team for their effort. Thanks are especially due to the Gateshead Millennium Study families and children for their participation in the study.

FUNDING

For peer review only

This work was supported by the National Prevention Research Initiative (NPRI) grant number GO 501306.

COMPETING INTERESTS

None declared.

For peer review only

REFERENCES

1. Department of Health. Healthy lives, healthy people: A call to action on obesity in England. London, 2011.

2. www.letsmove.gov. Let's Move! , 2010,pWebsite for Michelle Obama's campaign to reduce childhood obesity.

3. The Health and Social Care Information Centre. National Child Measurement Programme: England, 2011/2012 school year. 2012.

4. Chief Medical Officers of England Scotland Wales and Northern Ireland. Start active, stay active: a report on physical activity from the four home countries' Chief Medical Officers. Department of Health: London, 2011.

5. Strong WB, Malina RM, Blimkie CJR*, et al.* Evidence based physical activity for school-age youth. *J Pediatr* 2005;**146:**732-737.

6. Health Survey for England 2008. Volume 1: Physical activity and fitness. In: Craig R, Mindell J, Hirani V (eds), 2009.

7. NHS Choices. Healthy Eating. 2013.

8. Department of Health. National Diet and Nutrition Survey. Headline results from Years 1 and 2 (combined) of the Rolling Programme (2008/2009 - 2009/2010). In: Bates B, Lennox A, Bates C, Swan G (eds). HMSO: London, 2011.

For peer review only

9. Janz KF, Kwon S, Letuchy EM*, et al.* Sustained Effect of Early Physical Activity on Body Fat Mass in Older Children. *Am J Prev Med* 2009;**37:**35-40.

10. Must A, Barish EE, Bandini LG. Modifiable risk factors in relation to changes in BMI and fatness: what have we learned from prospective studies of school-aged children[quest]. *Int J Obes* 2009;**33:**705-715.

11. Craig LCA, McNeill G, Macdiarmid JI*, et al.* Dietary patterns of school-age children in Scotland: association with socio-economic indicators, physical activity and obesity. *Br J Nutr* 2009;**103:**319-334.

12. Basterfield L, Adamson AJ, Parkinson KN*, et al.* Surveillance of physical activity in the UK is flawed: Validation of the Health Survey for England Physical Activity Questionnaire. *Arch Dis Child* 2008;**93:**1054-1058.

13. Parkinson KN, Pearce MS, Dale A*, et al.* Cohort Profile: The Gateshead Millennium Study. *Int J Epidemiol* 2010;**40:**308-317.

14. Reilly JJ, Jackson DM, Montgomery C*, et al.* Total energy expenditure and physical activity in young Scottish children: mixed longitudinal study. *Lancet* 2004;**363:**211-212.

15. Reilly JJ, Penpraze V, Hislop J*, et al.* Objective measurement of physical activity and sedentary behaviour: Review with new data. *Arch Dis Child* 2008;**93:**614-619.

16. Puyau MR, Adolph AL, Vohra FA*, et al.* Validation and calibration of physical activity monitors in children. *Obes Res* 2002;**10:**150-157.

For peer review only

17. Reilly JJ, Coyle J, Kelly L*, et al.* An objective method for measurement of sedentary behavior in 3- to 4-year olds. *Obes Res* 2003;**11:**1155-1158.

18. Corder K, Brage S, Ramachandran A*, et al.* Comparison of two Actigraph models for assessing free-living physical activity in Indian adolescents. *J Sports Sci* 2007;**25:**1607-1611.

19. Basterfield L, Adamson AJ, Pearce MS*, et al.* Stability of habitual physical activity and sedentary behavior monitoring by accelerometry in 6-8 year olds. *J Phys Act Health* 2011;**8:**543-547.

20. Adamson AJ, Griffiths JM, Carlin LE*, et al.* FAST: Food Assessment in Schools Tool. *Proc Nutr Soc* 2003;**62:**84A.

21. Gregory JR, Lowe S, Bates CJ*, et al.* National Diet and Nutrition Survey: Young People Aged 4 to 18 Years. Volume 1: Report of the Diet and Nutrition Survey. The Stationary Office: London, 2000.

22. Cole TJ, Bellizzi MC, Flegal KM*, et al.* Establishing a standard definition for child overweight and obesity worldwide: international survey. *Br Med J* 2000;**320:**1240-1243.

23. Cole TJ, Flegal KM, Nicholls D*, et al.* Body mass index cut offs to define thinness in children and adolescents: international survey. *Br Med J* 2007;**335:**194-197.

For peer review only

24. Trost SG, Loprinzi PD, Moore R, *et al.* Comparison of Accelerometer Cut Points for Predicting Activity Intensity in Youth. *Med Sci Sports Exerc* 2011;**43:**1360-1368.

25. Riddoch CJ, Mattocks C, Deere K, *et al.* Objective measurement of levels and patterns of physical activity. *Arch Dis Child* 2007;**92:**963-969.

26. Riddoch CJ, Andersen LB, Wedderkopp N, *et al.* Physical activity levels and patterns of 9-and 15-yr-old European children. *Med Sci Sports Exerc* 2004;**36:**86-92.

27. Martinez-Gomez D, Ruiz JR, Ortega FB, *et al.* Recommended Levels of Physical Activity to Avoid an Excess of Body Fat in European Adolescents The HELENA Study. *Am J Prev Med* 2010;**39:**203-211.

28. Apete GK, Zitouni D, Hubert H, *et al.* Compliance of children in northern France with physical activity recommendations. *Perspect Public Health* 2012;**132:**81-88.

29. Jago R, Wedderkopp N, Kristensen PL, *et al.* Six-Year Change in Youth Physical Activity and Effect on Fasting Insulin and HOMA-IR. *Am J Prev Med* 2008;**35:**554-560.

30. Basterfield L, Adamson AJ, Frary JK, *et al.* Longitudinal study of physical activity and sedentary behavior in children. *Pediatrics* 2011;**127:**E24-E30.

31. Basterfield L, Pearce MS, Adamson AJ, *et al.* Physical activity, sedentary behavior, and adiposity in English children. *Am J Prev Med* 2012;**42:**445-451.

For peer review only

32. Ruxton CHS, Derbyshire E. Diet adequacy in UK schoolchildren. *Nutrition & Food Science* 2011;**41:**20-33.

33. Fremeaux AE, Hosking J, Metcalf BS*, et al.* Consistency of children's dietary choices: annual repeat measures from 5 to 13 years (EarlyBird 49). *Br J Nutr* 2011;**106:**725-731.

34. Jago R, Ness AR, Emmett P*, et al.* Obesogenic diet and physical activity: independent or associated behaviours in adolescents? *Public Health Nutr* 2010;**13:**673-681.

35. Papandreou D, Malindretos P, Rousso I. Risk factors for childhood obesity in a Greek paediatric population. *Public Health Nutr* 2010;**First View:**1-5.

36. Ochoa MC, Moreno-Aliaga MJ, Martínez-González MA*, et al.* Predictor factors for childhood obesity in a Spanish case-control study. *Nutrition* 2007;**23:**379-384.

37. Lagiou A, Parava M. Correlates of childhood obesity in Athens, Greece. *Public Health Nutr* 2008;**11:**940-945.

38. Lioret S, Touvier M, Lafay L*, et al.* Dietary and Physical Activity Patterns in French Children Are Related to Overweight and Socioeconomic Status. *J Nutr* 2008;**138:**101-107.

39. Garipagaoglu M, Budak N, Süt N*, et al.* Obesity risk factors in Turkish children. *J Pediatr Nurs* 2009;**24:**332-337.

For peer review only - http://bmjopen.bmj.com/site/about/guidelines.xhtml

AUTHOR CONTRIBUTIONS

AJA and KNP were responsible for the study conception and design, manuscript drafting and revision. AJA is the study guarantor. JJR and MSP were responsible for the study conception and design, analysis and interpretation of data, manuscript drafting and revision. LB and ARJ were responsible for data acquisition, analysis and interpretation of data; manuscript drafting and revision. All authors have had full access to all of the data in the study and take responsibility for the integrity of the data and the accuracy of the data analysis

For peer review only

STROBE Statement—Checklist of items that should be included in reports of ***cross-sectional studies***

	Item No	Whether included	Page or table in submitted manuscript	Article and page of uploaded supplementary material	Recommendation
**Title and abstract**	1	YES	Page 1: Title		(*a*) Indicate the study's design with a commonly used term in the title or the abstract
		YES	Page 2-3: Abstract		(*b*) Provide in the abstract an informative and balanced summary of what was done and what was found
**Introduction**					
Background/rationale	2	YES	Page 4: Introduction		Explain the scientific background and rationale for the investigation being reported
Objectives	3	YES	Page 5: Introduction	Gateshead Millennium Study protocol Upload 1 Page 3-4	State specific objectives, including any prespecified hypotheses
**Methods**					
Study design	4	YES	Page 5, 6 & 7: Methods Participants section Measures section	Gateshead Millennium Study protocol Upload 1 Page 4-6	Present key elements of study design early in the paper
Setting	5	YES	Page 5: Methods Participants section	Gateshead Millennium Study protocol Upload 1 Page 4-6	Describe the setting, locations, and relevant dates, including periods of recruitment, exposure, follow-up, and data collection
Participants	6	YES	Page 5: Methods Participants section	Gateshead Millennium Study protocol Upload 1 Page 4 Parkinson et al (2011) Int J Epi – Cohort Profile.	(*a*) Give the eligibility criteria, and the sources and methods of selection of participants

For peer review only - http://bmjopen.bmj.com/site/about/guidelines.xhtml

Variables	7	YES	Page 5-7: Methods Measures section	Parkinson et al (2011) Int J Epi – Cohort Profile.	Clearly define all outcomes, exposures, predictors, potential confounders, and effect modifiers. Give diagnostic criteria, if applicable
Data sources/ measurement	8*	YES	Page 5-7: Methods	Gateshead Millennium Study protocol<br>Upload 1 Page 4	For each variable of interest, give sources of data and details of methods of assessment (measurement). Describe comparability of assessment methods if there is more than one group
Bias	9	YES		Parkinson et al (2011) Int J Epi – Cohort Profile	Describe any efforts to address potential sources of bias
Study size	10	YES	Page 7: Methods Statistical analysis section		Explain how the study size was arrived at
Quantitative variables	11	YES	Page 7: Methods Statistical analysis section		Explain how quantitative variables were handled in the analyses. If applicable, describe which groupings were chosen and why
Statistical methods	12	YES	Page 7: Methods Statistical analysis section		(*a*) Describe all statistical methods, including those used to control for confounding
		YES	Page 7: Methods Statistical analysis section		(*b*) Describe any methods used to examine subgroups and interactions
		YES	Minimal; page 8: Results section first paragraph		(*c*) Explain how missing data were addressed
		N/A			(*d*) If applicable, describe analytical methods taking account of sampling strategy
		N/A			(*e*) Describe any sensitivity analyses
**Results**					
Participants	13*	YES	Page 8: Results	Parkinson et al (2011) Int J Epi – Cohort Profile.	(a) Report numbers of individuals at each stage of study—eg numbers potentially eligible, examined for eligibility, confirmed eligible, included in the study, completing follow-up, and analysed
		NO		Parkinson et al (2011) Int J Epi – Cohort Profile.	(b) Give reasons for non-participation at each stage
		N/A			(c) Consider use of a flow diagram

For peer review only

Descriptive data	14*	YES	Table 1	(a) Give characteristics of study participants (eg demographic, clinical, social) and information on exposures and potential confounders
		YES	Page 8: Results	(b) Indicate number of participants with missing data for each variable of interest
Outcome data	15*	YES	Tables 2, 3, 4.	Report numbers of outcome events or summary measures
Main results	16	YES	Page 16: Results<br>Table 5	(*a*) Give unadjusted estimates and, if applicable, confounder-adjusted estimates and their precision (eg, 95% confidence interval). Make clear which confounders were adjusted for and why they were included
		YES	Page 7	(*b*) Report category boundaries when continuous variables were categorized
		Not relevant		(*c*) If relevant, consider translating estimates of relative risk into absolute risk for a meaningful time period
Other analyses	17	YES	Page 16<br>Results	Report other analyses done—eg analyses of subgroups and interactions, and sensitivity analyses
**Discussion**				
Key results	18	YES	Page 16: Discussion<br>Paragraph 1	Summarise key results with reference to study objectives
Limitations	19	YES	Pages 16: Discussion<br>Paragraph 2	Discuss limitations of the study, taking into account sources of potential bias or imprecision. Discuss both direction and magnitude of any potential bias
Interpretation	20	YES	Pages 16 - 19: Discussion	Give a cautious overall interpretation of results considering objectives, limitations, multiplicity of analyses, results from similar studies, and other relevant evidence
Generalisability	21	YES	Pages 16 – 19: Discussion	Discuss the generalisability (external validity) of the study results
**Other information**				
Funding	22	YES	Page 20	Give the source of funding and the role of the funders for the present study and, if applicable, for the original study on which the present article is based

For peer review only - http://bmjopen.bmj.com/site/about/guidelines.xhtml

For peer review only

*Give information separately for exposed and unexposed groups.

Note: An Explanation and Elaboration article discusses each checklist item and gives methodological background and published examples of transparent reporting. The STROBE checklist is best used in conjunction with this article (freely available on the Web sites of PLoS Medicine at http://www.plosmedicine.org/, Annals of Internal Medicine at http://www.annals.org/, and Epidemiology at http://www.epidem.com/). Information on the STROBE Initiative is available at www.strobe-statement.org.

For peer review only

BMJ Open

Physical activity, diet and BMI in 6-8 year old children; a cross-sectional analysis

Journal:	*BMJ Open*
Manuscript ID:	bmjopen-2014-005001.R1
Article Type:	Research
Date Submitted by the Author:	17-Apr-2014
Complete List of Authors:	Basterfield, Laura; Newcastle University, Institute of Health & Society<br>Jones, Angela; Newcastle University, Institute of Health & Society<br>Parkinson, Kathryn; Newcastle University, Institute of Health & Society<br>Reilly, Jessica; Newcastle University, Institute of Health & Society<br>Pearce, Mark; Newcastle University, Institute of Health & Society<br>Reilly, John; University of Strathclyde,<br>Adamson, Ashley; Newcastle University, Institute of Health & Society
**Primary Subject Heading**:	Paediatrics
Secondary Subject Heading:	Public health
Keywords:	Community child health < PAEDIATRICS, PUBLIC HEALTH, EPIDEMIOLOGY

SCHOLARONE™ Manuscripts

For peer review only

Physical activity, diet and BMI in 6-8 year old children; a cross-sectional analysis

Corresponding author:

Ashley J Adamson, Institute of Health & Society, Human Nutrition Research Centre, Newcastle University, Newcastle upon Tyne, UK NE2 4HH.

Ashley.Adamson@ncl.ac.uk, Tel: 0191 208 5276, Fax: 0191 208 6043.

Co-authors:

Laura Basterfield, Institute of Health & Society, Human Nutrition Research Centre, Newcastle University, Newcastle upon Tyne, UK

Angela R Jones, Institute of Health & Society, Human Nutrition Research Centre, Newcastle University, Newcastle upon Tyne, UK

Kathryn N Parkinson, Institute of Health & Society, Human Nutrition Research Centre, Newcastle University, Newcastle upon Tyne, UK

Jessica Reilly, Institute of Health & Society, Human Nutrition Research Centre, Newcastle University, Newcastle upon Tyne, UK Mark S Pearce, Institute of Health & Society, Newcastle University, Sir James Spence Institute, Royal Victoria Infirmary, Newcastle upon Tyne, UK

John J Reilly, Physical Activity for Health Group, School of Psychological Sciences and Health, University of Strathclyde, Glasgow, UK

The Gateshead Millennium Study Core Team: Ashley Adamson, Anne Dale, Robert Drewett, Ann Le Couteur, Paul McArdle, Kathryn Parkinson, Mark Pearce, John Reilly, Charlotte Wright

Key words: epidemiologic studies; child; overweight; physical activity; diet

Word count: 3702

For peer review only

For peer review only - http://bmjopen.bmj.com/site/about/guidelines.xhtml

ABSTRACT

Objective

To assess relationships between current physical activity (PA), dietary intakeand body mass index (BMI) in English children.

Design and setting

Longitudinal birth cohort study in northeast England, cross-sectional analysis.

Participants

425 children (41% of the original cohort) aged 6-8 years (49% boys).

Main outcome measures

PA over seven days was measured objectively by accelerometer, three categories of PA were created: 'Active' ≥60min/d moderate-to-vigorous-intensity PA (MVPA); 'moderately active' 30-59min/d MVPA; inactive <30min/d MVPA. Dietary intake over four days was measured using a prospective dietary assessment tool which incorporated elements of both the food diary and food frequency methods. Three diet categories were created: 'healthy'; 'unhealthy' and 'mixed', according to the number of portions of different foods consumed. Adherence to the '5 a day' recommendations for portions of fruit and vegetables was also assessed. Children were classified as 'healthy weight' or 'overweight or obese' (OW/OB) according to International Obesity Taskforce cutpoints for BMI. Associations between weight status and PA/diet categories were analysed using logistic regression.

Results

Few children met the UK recommended guidelines for either MVPA or fruit and vegetable intake, with just 7% meeting the recommended amount of MVPA of 60 mins/d, and 3% meeting the 5-a-day fruit and vegetable recommendation. Higher PA was associated with a lower odds ratio for OW/OB in boys only (0.20 95% CI 0.04-0.88). There was no association detected between dietary intake and OW/OB in either sex.

Conclusions

For peer review only

Increasing MVPA may help to reduce OW/OB in boys; however, more research is required to examine this relationship in girls. Children are not meeting the UK guidelines for diet and PA, and more needs to be done to improve this situation.

Strengths and limitations of this study

- Direct objectively measured physical activity and observed dietary intake
- Direct measures of body composition
- Population based birth cohort socioeconomically representative of northeast England
- Ethnically homogeneous sample which reduces generalisability
- Cross-sectional so cannot determine direction of causality

For peer review only

INTRODUCTION

Childhood obesity is a major public health challenge both in the UK and around the world, with governments increasingly involving themselves in different strategies to reduce excess weight in the population.[1, 2] Recent findings from the National Child Measurement Programme (2011-2012) regarding the prevalence of childhood overweight and obesity are concerning,[3] especially in northeast England, which is the worst-affected region of England outside of London, where 24.5% of 4-5 year olds and 37.0% of 10-11 year olds are overweight or obese. There remains a limited understanding of the timing and causes of childhood obesity and there is an urgent need for effective preventive strategies.

Current UK health recommendations state that children should accumulate at least 60 minutes of moderate-vigorous intensity physical activity (MVPA) per day, with vigorous intensity activities performed on at least 3 days per week.[4] This recommendation is based on evidence from intervention studies.[5] However, recent evidence suggests that the majority of children fail to meet this target, with 51% of English 4-10 year old boys and 34% of 4-10year old girls achieving the physical activity recommendations as measured by accelerometer, figures which dropped to 7% and 0% among 11-15 year olds.[6] In addition, children should consume a healthy balanced diet which contains a variety of foods, including at least 5 portions of fruit and vegetables per day, plenty of starchy foods (particularly wholegrain varieties), some protein-rich foods and some milk and dairy foods. It is also recommended that foods high in fat (especially saturated fat), salt and sugar should only be consumed in small amounts.[7] Data from the National Diet and Nutrition Survey in 2010 indicated that children aged 4-10 years consume an average of 173g of fruit and vegetables daily, equivalent to 2.2 80g portions (or 2.8 including fruit juice).[8] Data from longitudinal cohorts are beginning to uncover the associations between low levels of physical activity and unfavourable body composition e.g. Janz et al (2009),[9] but the data on the relationship between diet/dietary components and body composition have so far been equivocal, as reviewed by Must et al.[10] This may be due to the contrasting methods of dietary assessment and analysis and the complexity of dietary patterns; given the importance of energy balance in maintenance of a healthy body weight it is vital that measurement of dietary intake continues to be improved. Evidence of the impact of *both* objectively measured physical activity (PA)/sedentary behaviour (SB) *and* food intake on body composition in primary school-age children is lacking, with most studies measuring either physical

For peer review only - http://bmjopen.bmj.com/site/about/guidelines.xhtml

For peer review only

activity *or* food intake. A recent Scottish study assessed the effect of dietary patterns and physical activity on body mass index (BMI),[11] but used a questionnaire that is known to overestimate time spent in physical activity.[12] The authors did not find a consistent association between dietary patterns or time spent in physical activity and BMI group.[11]

The primary aim of the present study was to describe the relationship between overweight and obesity, BMI, PA, and dietary habits in a sample of children that are socioeconomically representative of northeast England.[13]

METHODS

Participants

The Gateshead Millennium Study (GMS) cohort originally consisted of 1029 babies and their families and is described in detail elsewhere.[13] Briefly, all mothers resident in Gateshead who gave birth within pre-specified recruiting weeks between June 1999 and May 2000 were invited to take part, 81% (n=1011) agreed. Participants were mostly from the white ethnic majority (98%). Data collection for the present study involved a variety of lifestyle and anthropometric measures, and took place between October 2006 and December 2007 when the children were aged 6-8 years. The data presented are cross-sectional from the 6-8 year collection phase of the GMS. Ethical approval was given by Gateshead and South Tyneside Local National Health Service Research Ethics Committee. Parents provided informed written consent for their child's participation and children gave their written assent.

Measures

Physical activity and sedentary behaviour

PA and SB were objectively measured using Actigraph GT1M accelerometers (Actigraph LLC, Pensacola, Florida, USA) attached to an elastic belt and worn on the right hip as described previously.[14] Children were asked to wear the accelerometer during waking hours for 7 days and to remove them only for water-based activities. Parents were asked to complete a time-log detailing when the accelerometer had been worn.

For peer review only

The accelerometers collected data in 15 second sampling intervals (epochs); prior to analysis the data were collapsed to 60 second epochs. Data were verified manually using the time-log and accelerometry output as described previously[12] to delete occasional periods of non-wear time.

Three constructs of PA and one of SB were generated: total volume of activity (expressed as mean daily accelerometer counts per minute, cpm); MVPA daily, equivalent to energy expenditure in excess of approximately three times resting energy expenditure (expressed as both minutes per day and % of daily time spent in MVPA), and sedentary behaviour (equivalent to no translocation of the trunk[15] (expressed as % daily time spent in SB)).

The cutpoint of ≥3200 cpm[16] was used to define MVPA; and <1100 cpm[17] was used to define SB. The Actigraph GT1M model has been shown to have a consistent 9% bias relative to model 7164[18] from which the cutpoints were derived, so a +9% correction was applied to the raw accelerometry data before applying cutpoints. Of 606 accelerometers given out, 510 were worn for at least 3 days and for at least 6 hours per day[19] and thus were deemed eligible for analysis in the present study.

Dietary intake

Food intake in the children was assessed over 4 days (2 week days and 2 weekend days) using the 'Food Assessment in Schools Tool' (FAST) food diary.[20] The FAST food diary is a prospective dietary assessment method which incorporates elements of both the food diary and the food frequency methods and was specifically designed to assess the diets of primary school children. Briefly, each day is split into six timeslots, each containing a simple tick list of up to 50 foods commonly eaten by children of this age.[21] Space for additional foods was also provided. Researchers and lay-observers recorded food intake for each child during the school day, and parents and other carers recorded food intake at all other times. No interviews or estimations of portion size were required during data collection, average age- and sex- specific portion sizes for all foods consumed were ascertained using relevant UK National Diet and Nutrition Survey data.[20, 21] This method allowed analysis of food choice at individual level but not of energy intake.

A 'healthy' and 'unhealthy' dietary indicator score was created for each of the four days, and a daily mean calculated. The 'healthy' indicator (HI) score was calculated by summing the number of portions consumed of the following foods: wholemeal bread; non-high sugar wholegrain breakfast cereals; fruit; vegetables; semi-skimmed

For peer review only - http://bmjopen.bmj.com/site/about/guidelines.xhtml

milk as a drink; semi-skimmed milk; non-processed meat and fish products; beans and pulses; water and white bread with added fibre. The 'unhealthy' indicator (UI) score was calculated by summing the number of portions consumed of the following foods: white bread; high-sugar refined breakfast cereals; fried chips; biscuits; confectionery, cakes and sweet puddings; full-fat milk as a drink; full-fat milk; processed meat and fish products; full sugar cordial or squash; full sugar carbonated drinks; chocolate/milkshake powder; crisps and savoury snacks and sugar. A cereal was categorised as 'non-high sugar' if it contained <5g sugar/100g and 'high sugar' if it contained >15g sugar/100g as manufactured. The addition of discretionary sugar was not taken into account when categorising cereals. To assess adherence to dietary guidelines, fruit and vegetable consumption was measured in 80g amounts, fruit juice in 150ml portions. Only one portion of fruit juice may count towards daily fruit and vegetable intake.[7]

Anthropometry

All body measurements were taken in duplicate with the children in light indoor clothing, without socks and shoes. Height was measured to 0.1cm with a Leicester portable height measure (Chasmors, London, UK), waist to 0.1cm at the minimum circumference between the lowest rib and iliac crest, and weight to 0.1kg using a TANITA TBF 300MA body fat analyser (Chasmors, London, UK). BMI was calculated (wt (kg)/ht(m^2)). Categories of underweight, healthy weight, overweight and obesity were calculated according to age- and sex-specific International Obesity Taskforce (IOTF) criteria.[22, 23]

Statistical analysis

The sample size for the current cross-sectional study was fixed by the size of the cohort, participant attrition and those children providing complete data for PA, dietary intake and anthropometry at this data sweep.

Analysis was performed using SPSS 17 and STATA 10. Shapiro-Wilk tests assessed the normality of distribution of variables. As data were skewed, Mann-Whitney tests were performed to test for differences between sexes and Wilcoxon Rank Tests to assess differences in weekend and weekday PA/SB. Median and interquartile ranges are reported unless otherwise stated.

Logistic regression was used to predict the likelihood of being overweight or obese (OW/OB) at different levels of PA and with different dietary indicator scores. Children were assigned to one of three PA categories: (1) 'active': those who met the ≥60 minutes per day guidelines (this figure was generated by dividing the total amount of

For peer review only

MVPA for the week by the number of days the accelerometer was worn.); (2) 'moderately active': those who did between 30 and 60 min·d^{-1}; (3) 'inactive': those who did less than 30 min·d^{-1}. Children were also assigned to one of three dietary categories: (1) 'healthy': those children whose HI score was ≥ the median and whose UI score was < the median ; (2) 'unhealthy': those children whose UI score was ≥ the median and whose HI score < the median ; (3) 'mixed': those diets which neither met the criteria for a 'healthy' diet or 'unhealthy' diet. Children were lastly classified by BMI as: (1) not overweight or obese; (2) overweight or obese. Odds ratios (OR) and corresponding 95% confidence intervals (95% CI) are reported. Significance was set at p<0.05. Sex was included in the regression analysis as a potential confounder. Townsend score from birth (a measure of deprivation) was unrelated to any of the variables and thus not included in the final analysis.

RESULTS

Overall, 510 children completed sufficient accelerometry recording, 474 children returned completed FAST food diaries and 597 children had anthropometry measurements. Data from all three domains were provided by 425 children and were used in the analysis. There were no significant differences in food intake, PA or BMI between children providing complete records and those who did not. Children had a mean age of 7.4y (range 6.4-8.4). Characteristics of the sample are displayed in Table 1.

For peer review only

Table 1 Participant characteristics (median and IQR), by sex

	Combined* (n=425)		Boys (n=211) (49.6%)		Girls (n=214) (50.4%)		p-value
Height (cm)	1.24	1.21, 1.29	1.26	1.22, 1.29	1.24	1.21, 1.28	*0.039*
Weight (kg)	26.4	22.7, 28.7	25.7	23.0, 29.1	25.0	22.5, 28.4	0.264
Waist (cm)	55.7	52.6, 59.0	56.5	53.5, 59.4	54.8	52.1, 58.7	*0.006*
BMI (kg/m^2)	16.2	15.3, 17.8	16.1	15.4, 17.8	16.3	15.2, 17.8	0.878

*n for individual measures varies from 421-425. Mann-Whitney tests performed. BMI, body mass index

For peer review only - http://bmjopen.bmj.com/site/about/guidelines.xhtml

Using IOTF cutoffs,[22] 24% of the children were overweight or obese, with no significant difference between girls and boys (Mann-Whitney test, p=0.885). The full distribution of children between weight categories is shown in Table 2.

Table 2 Distribution of children between weight categories, by sex

	Combined		Boys (49.6%)		Girls (50.4%)	
	n	%	n	%	n	%
Underweight	3	0.7	1	0.5	2	0.9
Healthy weight	320	75.3	160	75.8	160	74.8
Overweight	75	17.6	35	16.6	40	18.7
Obese	27	6.4	15	7.1	12	5.6
Total	425	100	211	100	214	100

There were no significant differences between the sexes in the number of days the activity belt was worn or the total volume of activity (mean cpm) (Table 3).

For peer review only

Table 3 Comparison of physical activity, sedentary behaviour and dietary intake between sexes (median and IQR unless otherwise specified)

	Combined (n=425)		Boys (n=211)		Girls (n=214)		p-value
Physical activity/sedentary behaviour							
Number of days accelerometer worn	7	6,7	7	6,7	7	6,7	0.380
Hours worn/d	11.3	10.6, 11.9	11.4	10.7, 12.0	11.1	10.4, 11.8	0.077
Mean cpm/d	727	604, 878	740	624, 881	718	591, 868	0.313
MVPA (min/d)	26	18, 38	28	19, 43	25	16, 36	*0.020*
% time in MVPA	3.9	2.6, 5.7	4.2	2.7, 6.3	3.8	2.5, 5.5	*0.043*
% time SB	77.7	73.6, 81.8	76.9	71.9, 80.8	78.4	74.6, 82.7	*0.002*
Achieved 60 min/d MVPA (n, %)*	31	7.3	23	10.9	8	3.7	*0.005*
Dietary intake							
Portions of fruit & vegetables consumed/d	1.7	1.1, 2.5	1.7	1.0, 2.5	1.8	1.2, 2.5	0.211
Portions of fruit & vegetables consumed/d$	2.1	1.4,3.0	2.0	1.3,2.9	2.2	1.4,3.1	0.224
Ate 5 portions of fruit & vegetables/d (n, %)$	13	3.1	8	3.8	5	2.3	0.384
Number of healthy indicator foods/d	5.0	3.8, 6.5	5.0	3.8, 6.5	5.1	3.8, 6.5	0.342
Number of unhealthy indicator foods/d	5.3	4.3, 6.3	5.5	4.3, 6.3	5.3	4.3, 6.3	0.496

MVPA, moderate-vigorous intensity physical activity

*total MVPA for the week/days worn, $including one 150ml portion of juice. Mann-Whitney tests performed.

For peer review only - http://bmjopen.bmj.com/site/about/guidelines.xhtml

Levels of activity in general were low, as reported previously; [12] only 31 children (7%) achieved an average of at least 60 min MVPA·d^{-1}, 23 boys and 8 girls. Only two children (0.5%) accumulated at least 60 minutes on every day they wore the accelerometer. The majority of time was spent in sedentary behaviour (median 78% of wear time, equivalent to around 8.6 hours per day). When looking at the differences between weekend and weekday activity, volume of activity (cpm) was similar, and slightly but significantly more MVPA was taken on weekdays ($p<0.0001$) (data not shown). Girls had a greater volume of activity at the weekend but a lower time spent in MVPA, suggesting the majority of their activities are in the sedentary or light intensity categories. Boys spent significantly more time in MVPA on weekdays than girls.

Dietary intake was similar for boys and girls (Table 3). Children consumed a median of 1.7 portions of fruit and vegetables daily (or 2.1 portions when including fruit juice), with just 13/425 children (3.1%) consuming their '5 a day'. On average, children ate 5.0 healthy indicator foods and 5.3 unhealthy indicator foods daily.

Table 4 displays the distribution of overweight/obesity by diet and activity group.

For peer review only

Table 4. Distribution of OW/OB by physical activity and dietary category (n,%)

	Healthy weight (323, 76.0)				Overweight or obese (102, 24.0)			
	Dietary Pattern				Dietary Pattern			
PA category	Unhealthy	Mixed	Healthy	Total	Unhealthy	Mixed	Healthy	Total
**Boys** (211, 49.6)								
Inactive	27, 16.8	33, 20.5	18, 11.2	78, 48.4	11, 22.0	13, 26.0	14, 28.0	38, 76.0
Moderately active	17, 10.6	28, 17.4	17, 10.6	62, 38.5	2, 4.0	7, 14.0	1, 2.0	10, 20.0
Active	9, 5.6	10, 6.2	2, 1.2	21, 13.0	0, 0.0	1, 2.0	1, 2.0	2, 4.0
Total	53, 32.9	71, 44.1	37, 23.0	161, 100.0	13, 26.0	21, 42.0	16, 32.0	50, 100.0
**Girls** (214, 50.4)								
Inactive	27, 16.7	29, 17.9	40, 24.7	96, 59.3	9, 17.3	18, 34.6	10, 19.2	37, 71.2
Moderately active	17, 10.5	31, 19.1	12, 7.4	60, 37.0	4, 7.7	4, 7.7	5, 9.6	13, 25.0
Active	1, 0.6	3, 1.9	2, 1.2	6, 3.7	0, 0.0	2, 3.8	0, 0.0	2, 3.8
Total	45, 27.8	63, 38.9	54, 33.3	162, 100.0	13, 25.0	24, 46.2	15, 28.8	52, 100.0

For peer review only

For peer review only - http://bmjopen.bmj.com/site/about/guidelines.xhtml

13% of healthy weight boys were in the ‘active’ PA category, compared with just 4% of OW/OB boys. 48% of healthy weight boys were inactive compared with 76% of OW/OB boys. There was less variation for girls, as only 8 girls in total were in the active category. 59% of healthy weight girls were in the inactive category compared with 71% of OW/OB girls.

Compared with the reference group (inactive), children in the moderately active group had a significantly lower risk of being OW/OB (OR=0.44, 0.26-0.74 (Table 5)).

For peer review only

For peer review only - http://bmjopen.bmj.com/site/about/guidelines.xhtml

Table 5 Likelihood of being overweight or obese for different activity levels and dietary patterns (odds ratios and 95% CI)

	Combined (n=425)	p-value	Boys (n=211)	p-value	Girls (n=214)	p-value
Inactive (reference)	1		1		1	
Moderately active	0.44 (0.26-0.74)	*0.002*	0.33 (0.15-0.72)	*0.005*	0.56 (0.28-1.14)	0.112
Active	0.34 (0.12-1.02)	0.054	0.20 (0.04-0.88)	*0.033*	0.86 (0.17-4.48)	0.863
Unhealthy diet (reference)	1		1		1	
Mixed diet	1.27 (0.73-2.19)	0.40	1.21 (0.55-2.62)	0.637	1.32 (0.61-2.86)	0.485
Healthy diet	1.28 (0.71-2.33)	0.41	1.76 (0.76-4.10)	0.188	0.96 (0.42-2.23)	0.927

For peer review only - http://bmjopen.bmj.com/site/about/guidelines.xhtml

For peer review only

Results for children in the active group (≥60 min MVPA·d^{-1} (OR=0.34, 95% CI 0.12-1.02) approached significance (p=0.054). Similar results were seen after adjusting for sex. When analysing girls and boys separately, the association between PA and risk of OW/OB remained only for boys. Boys who were moderately active had an OR of 0.33 (0.15-0.72) for risk of OW/OB (*p=0.005*), and boys in the active category had an OR of 0.20 (0.04-0.88) (*p=0.033*), compared with the inactive group. Moderately active girls had an OR of 0.56 (0.28-1.14) (p=0.112) and active girls an OR of 0.86 (0.17-4.48) (p=0.863) for OW/OB compared with inactive girls. Similar results were seen for activity when adjusting for diet group.

There was no influence of dietary pattern on risk of OW/OB: OR 1.27 (0.73-2.19 p=0.40) for the mixed diet group, 1.28 (0.71-2.33) for the healthy group (p=0.41) compared with the reference group (unhealthy diet) (Table 5), with similar results after adjusting for sex.

There was no evidence of an interaction between PA and food intake on risk of OW/OB (p=0.444).

DISCUSSION

Our results provide evidence that children in the north east of England are not meeting the guidelines for physical activity and nutrition that are recommended for good health. Lack of moderate-vigorous intensity physical activity was associated with an increased risk of being overweight and obese in boys. The lack of an association in girls could be due to the low numbers being sufficiently active.

The present study has limitations in terms of the ethnically homogenous sample, which may limit the generalisability of the findings to other samples and settings. There was also a lack of variability in PA, with data skewed towards inactivity, particularly in girls. There are several cut-points in use for classifying MVPA[24] and the value of 3200cpm used in the current study was obtained through comparison with energy expenditure,[16] and similar to those from other studies.[15] Given the on-going discussion regarding the choice of cut-point there may be no suitable cut-point for all ages of childhood. As the data are cross-sectional, it is not possible to determine the direction of causality, which will be addressed by future follow up. However, the strengths of this study include the relatively large sample size, the focused age of the children, and the use of objective measures of PA (accelerometry) combined with dietary observations is unique.

For peer review only - http://bmjopen.bmj.com/site/about/guidelines.xhtml

For peer review only

Few other studies have used objective methods to describe levels of habitual physical activity and sedentary behaviour in 6-8 year olds in combination with dietary intake measures. Recent UK studies in 3-5 year olds[14] and 11-12 year olds[25] which have used the same objective methods of PA measurement have found similarly low levels of physical activity with adherence to the 60 minutes/day recommendation of MVPA being achieved in <5% of their samples. In contrast, UK studies which have used subjective methods (questionnaires) have generally reported much higher levels of MVPA, for example the Health Survey for England, which leads to over-estimates of PA.[12] Low levels of physical activity were present in our sample well before puberty, the period traditionally regarded as being associated with low levels of physical activity. In keeping with other studies[9], [26], we found that boys were more active than girls, and the lack of association of PA on risk of OW/OB in the girls compared with the boys suggests further work is required into the mechanisms by which increasing PA and/or decreasing SB reduces risk of OW/OB in children. Few studies have looked at the relationship between the '60min' guidelines and body composition. Martinez-Gomez et al (2010)[27] reported that 60 min·d^{-1} MVPA could discriminate between normal weight and OW/OB adolescents (mean age 14.7 years), however they used a lower cut-point for MVPA of 2000cpm, compared with the 3200cpm used in the present study. This resulted in average MVPA·d^{-1} of 66 minutes for boys and 50 minutes for girls.[27] Recent evidence using 3200cpm as the MVPA cutpoint from 10 year olds in France[28] found that only 10% of the 'normal weight' children reached 60 min·d^{-1}, but 44% did at least 30 min·d^{-1}. In addition, although only 5% of OW/OB children did at least 60min·d^{-1} MVPA, 34% reached 30 min·d^{-1}.[28] Our results show that this lower recommendation could still have benefits in terms of body composition, particularly for boys.

Whilst it is generally accepted that physical activity decreases with age,[29] pinpointing the age when the decrease starts is important for targeted interventions. Our results suggest that even at age 7 years physical activity levels are far lower than recommended. The great proportion of time spent sedentary by both sexes is concerning - in both sexes almost 7 hours per day were spent with no translocation of the trunk.[15] Both sexes took part in more MVPA on weekdays than weekends, highlighting both the importance the school environment plays in maintaining activity levels, and that more needs to be done to increase activity at the weekend. The cross-sectional nature of the present study means we cannot say whether the children have already started to reduce their activity. However, data we have published from the cohort at a 2 year follow-up show a decrease in MVPA from age 7

For peer review only

to 9 years, and the decrease in MVPA associated with an increase in BMI-z score in boys.[30, 31]

The low consumption of fruit and vegetables, and the relatively high consumption of an unhealthy diet in this sample are both cause for concern, despite the lack of association between the 'healthy' or 'unhealthy' categories and risk of OW/OB. The lack of association between dietary variables and risk of OW/OB in this study, as in others, may be due to the lack of individual portion sizes which prohibited the assessment of energy intake. The dietary assessment tool used in this study allowed direct observation of intake but applied standard portion sizes from a previous National Diet and Nutrition Survey which used a weighed intake method.[21] The amount for a 'portion' of fruits and vegetables was set at 80g, which is considered suitable for children aged over 11 years.[32] A lower amount may be more suitable for children under 11 years, but as this has not yet been identified,[32] 80g was used in this analysis. It is possible that overweight children are eating larger portions of healthy foods, or the parents are aware of their child's overweight and have reduced food intake accordingly. There may also be an element of mis-reporting or optimistic bias if the parent believes their child is overweight and eats either to excess or 'unhealthily'. Foods in the current study were assigned to the 'healthy' or 'unhealthy' group though a combination of current national guidelines for consumption and restriction[7] according to nutritional content. Since these analyses were performed, Frémeaux and colleagues[33] have published a longitudinal factor analysis of English children's food intake from age 5 to 13 years. They reported the emergence of two main diet types that matched almost identically with ours.[33] Recent evidence from Scottish 5-11 year olds did not find an association between dietary patterns and BMI or physical activity,[11] and observed an inverse association between 'screen time' and healthier eating patterns (diet measure with food frequency questionnaire, FFQ, and physical activity measured by questionnaire, PAQ). The ALSPAC cohort in the UK were assessed for associations between PA and diet at age 10-11 years, and found only weak relationships between the two[34] (3x1-day food diary at 10y, accelerometry at 11y). A Greek case-control study found a protective effect of PA[35] on risk of OW/OB, and an increased risk with increased time spent watching TV, and consumption of sugar-sweetened beverages (FFQ/PAQ), an observation also reported in Spanish children (FFQ/PAQ).[36] Others [37] found an effect of sedentary behaviour on risk of OW/OB, but again no effect of diet composition (questionnaire). A varied food pattern plus a physically active lifestyle was inversely related to OW in French children (7-day food diary/PAQ).[38] Similarly, reduced physical activity, but

For peer review only - http://bmjopen.bmj.com/site/about/guidelines.xhtml

not excessive energy intake, was associated with increased risk of OB in Turkish children (3-day food diary/PAQ).[39] However, these studies included a wide age range of children, and physical activity and sedentary behaviour were measured objectively only in the ALSPAC cohort[34], so our observations strengthen the evidence base for the protective effects of PA on risk of OW/OB.

In conclusion, children in northeast England have extremely low levels of habitual MVPA, high levels of sedentary behaviour and consume a diet low in fruits and vegetables but high in foods associated with an unhealthy diet. Increased PA was associated with reduced risk of OW/OB in boys. Further work is required to uncover the associations of PA/diet and OW/OB in girls and the longitudinal associations between PA/diet and OW/OB in boys and girls. These observations give cause for concern in relation to their current and future risk of cardiovascular diseases, diabetes, and obesity. Our observations suggest that UK government targets in relation to levels of physical activity and nutrition among children are not being met.

For peer review only

For peer review only - http://bmjopen.bmj.com/site/about/guidelines.xhtml

ACKNOWLEDGEMENTS

The Gateshead Millennium Study is supported by a grant from the National Prevention Research Initiative (incorporating funding from British Heart Foundation; Cancer Research UK; Department of Health; Diabetes UK; Economic and Social Research Council; Food Standards Agency; Medical Research Council; Research and Development Office for the Northern Ireland Health and Social Services; Chief Scientist Office, Scottish Government Health Directorates; Welsh Assembly Government and World Cancer Research Fund). The cohort was first established with funding from the Henry Smith Charity and Sport Aiding Research in Kids (SPARKS) and followed up with grants from Gateshead NHS Trust R&D, Northern and Yorkshire NHS R&D, and Northumberland, Tyne and Wear NHS Trust. We acknowledge the support of an External Reference Group in conducting the study. We appreciate the support of Gateshead Health NHS Foundation Trust, Gateshead Education Authority and local schools. We warmly thank the research team for their effort. Thanks are especially due to the Gateshead Millennium Study families and children for their participation in the study.

FUNDING

This work was supported by the National Prevention Research Initiative (NPRI) grant number GO 501306.

AUTHOR CONTRIBUTIONS

AJA and KNP were responsible for the study conception and design, manuscript drafting and revision. AJA is the study guarantor. JJR and MSP were responsible for the study conception and design, analysis and interpretation of data, manuscript drafting and revision. LB and ARJ were responsible for data acquisition, analysis and interpretation of data; manuscript drafting and revision. All authors have had full access to all of the data in the study and take responsibility for the integrity of the data and the accuracy of the data analysis

COMPETING INTERESTS

None declared.

DATA SHARING STATEMENT

Requests should be directed to the corresponding author.

For peer review only

REFERENCES

1. Department of Health. Healthy lives, healthy people: A call to action on obesity in England. London, 2011.

2. www.letsmove.gov. Let's Move! , 2010,pWebsite for Michelle Obama's campaign to reduce childhood obesity.

3. The Health and Social Care Information Centre. National Child Measurement Programme: England, 2011/2012 school year. 2012.

4. Chief Medical Officers of England Scotland Wales and Northern Ireland. Start active, stay active: a report on physical activity from the four home countries' Chief Medical Officers. Department of Health: London, 2011.

5. Strong WB, Malina RM, Blimkie CJR*, et al.* Evidence based physical activity for school-age youth. *J Pediatr* 2005;**146:**732-737.

6. Health Survey for England 2008. Volume 1: Physical activity and fitness. In: Craig R, Mindell J, Hirani V (eds), 2009.

7. NHS Choices. Healthy Eating. 2013.

8. Department of Health. National Diet and Nutrition Survey. Headline results from Years 1 and 2 (combined) of the Rolling Programme (2008/2009 - 2009/2010). In: Bates B, Lennox A, Bates C, Swan G (eds). HMSO: London, 2011.

For peer review only

9. Janz KF, Kwon S, Letuchy EM*, et al.* Sustained Effect of Early Physical Activity on Body Fat Mass in Older Children. *Am J Prev Med* 2009;**37:**35-40.

10. Must A, Barish EE, Bandini LG. Modifiable risk factors in relation to changes in BMI and fatness: what have we learned from prospective studies of school-aged children[quest]. *Int J Obes* 2009;**33:**705-715.

11. Craig LCA, McNeill G, Macdiarmid JI*, et al.* Dietary patterns of school-age children in Scotland: association with socio-economic indicators, physical activity and obesity. *Br J Nutr* 2009;**103:**319-334.

12. Basterfield L, Adamson AJ, Parkinson KN*, et al.* Surveillance of physical activity in the UK is flawed: Validation of the Health Survey for England Physical Activity Questionnaire. *Arch Dis Child* 2008;**93:**1054-1058.

13. Parkinson KN, Pearce MS, Dale A*, et al.* Cohort Profile: The Gateshead Millennium Study. *Int J Epidemiol* 2010;**40:**308-317.

14. Reilly JJ, Jackson DM, Montgomery C*, et al.* Total energy expenditure and physical activity in young Scottish children: mixed longitudinal study. *Lancet* 2004;**363:**211-212.

15. Reilly JJ, Penpraze V, Hislop J*, et al.* Objective measurement of physical activity and sedentary behaviour: Review with new data. *Arch Dis Child* 2008;**93:**614-619.

16. Puyau MR, Adolph AL, Vohra FA*, et al.* Validation and calibration of physical activity monitors in children. *Obes Res* 2002;**10:**150-157.

For peer review only

17. Reilly JJ, Coyle J, Kelly L*, et al.* An objective method for measurement of sedentary behavior in 3- to 4-year olds. *Obes Res* 2003;**11:**1155-1158.

18. Corder K, Brage S, Ramachandran A*, et al.* Comparison of two Actigraph models for assessing free-living physical activity in Indian adolescents. *J Sports Sci* 2007;**25:**1607-1611.

19. Basterfield L, Adamson AJ, Pearce MS*, et al.* Stability of habitual physical activity and sedentary behavior monitoring by accelerometry in 6-8 year olds. *J Phys Act Health* 2011;**8:**543-547.

20. Adamson AJ, Griffiths JM, Carlin LE*, et al.* FAST: Food Assessment in Schools Tool. *Proc Nutr Soc* 2003;**62:**84A.

21. Gregory JR, Lowe S, Bates CJ*, et al.* National Diet and Nutrition Survey: Young People Aged 4 to 18 Years. Volume 1: Report of the Diet and Nutrition Survey. The Stationary Office: London, 2000.

22. Cole TJ, Bellizzi MC, Flegal KM*, et al.* Establishing a standard definition for child overweight and obesity worldwide: international survey. *Br Med J* 2000;**320:**1240-1243.

23. Cole TJ, Flegal KM, Nicholls D*, et al.* Body mass index cut offs to define thinness in children and adolescents: international survey. *Br Med J* 2007;**335:**194-197.

For peer review only

24. Trost SG, Loprinzi PD, Moore R, *et al.* Comparison of Accelerometer Cut Points for Predicting Activity Intensity in Youth. *Med Sci Sports Exerc* 2011;**43:**1360-1368.

25. Riddoch CJ, Mattocks C, Deere K, *et al.* Objective measurement of levels and patterns of physical activity. *Arch Dis Child* 2007;**92:**963-969.

26. Riddoch CJ, Andersen LB, Wedderkopp N, *et al.* Physical activity levels and patterns of 9-and 15-yr-old European children. *Med Sci Sports Exerc* 2004;**36:**86-92.

27. Martinez-Gomez D, Ruiz JR, Ortega FB, *et al.* Recommended Levels of Physical Activity to Avoid an Excess of Body Fat in European Adolescents The HELENA Study. *Am J Prev Med* 2010;**39:**203-211.

28. Apete GK, Zitouni D, Hubert H, *et al.* Compliance of children in northern France with physical activity recommendations. *Perspect Public Health* 2012;**132:**81-88.

29. Jago R, Wedderkopp N, Kristensen PL, *et al.* Six-Year Change in Youth Physical Activity and Effect on Fasting Insulin and HOMA-IR. *Am J Prev Med* 2008;**35:**554-560.

30. Basterfield L, Adamson AJ, Frary JK, *et al.* Longitudinal study of physical activity and sedentary behavior in children. *Pediatrics* 2011;**127:**E24-E30.

31. Basterfield L, Pearce MS, Adamson AJ, *et al.* Physical activity, sedentary behavior, and adiposity in English children. *Am J Prev Med* 2012;**42:**445-451.

For peer review only

32. Ruxton CHS, Derbyshire E. Diet adequacy in UK schoolchildren. *Nutrition & Food Science* 2011;**41:**20-33.

33. Fremeaux AE, Hosking J, Metcalf BS*, et al.* Consistency of children's dietary choices: annual repeat measures from 5 to 13 years (EarlyBird 49). *Br J Nutr* 2011;**106:**725-731.

34. Jago R, Ness AR, Emmett P*, et al.* Obesogenic diet and physical activity: independent or associated behaviours in adolescents? *Public Health Nutr* 2010;**13:**673-681.

35. Papandreou D, Malindretos P, Rousso I. Risk factors for childhood obesity in a Greek paediatric population. *Public Health Nutr* 2010;**First View:**1-5.

36. Ochoa MC, Moreno-Aliaga MJ, Martínez-González MA*, et al.* Predictor factors for childhood obesity in a Spanish case-control study. *Nutrition* 2007;**23:**379-384.

37. Lagiou A, Parava M. Correlates of childhood obesity in Athens, Greece. *Public Health Nutr* 2008;**11:**940-945.

38. Lioret S, Touvier M, Lafay L*, et al.* Dietary and Physical Activity Patterns in French Children Are Related to Overweight and Socioeconomic Status. *J Nutr* 2008;**138:**101-107.

39. Garipagaoglu M, Budak N, Süt N*, et al.* Obesity risk factors in Turkish children. *J Pediatr Nurs* 2009;**24:**332-337.

For peer review only

For peer review only

P~~Impact of p~~hysical activity, ~~and~~ diet ~~on~~ and BMI in 6-8 year old children; a cross-sectional analysis

Corresponding author:
Ashley J Adamson, Institute of Health & Society, Human Nutrition Research Centre, Newcastle University, Newcastle upon Tyne, UK NE2 4HH.
Ashley.Adamson@ncl.ac.uk, Tel: 0191 208 5276, Fax: 0191 208 6043.

Co-authors:
Laura Basterfield, Institute of Health & Society, Human Nutrition Research Centre, Newcastle University, Newcastle upon Tyne, UK
Angela R Jones, Institute of Health & Society, Human Nutrition Research Centre, Newcastle University, Newcastle upon Tyne, UK
Kathryn N Parkinson, Institute of Health & Society, Human Nutrition Research Centre, Newcastle University, Newcastle upon Tyne, UK
Jessica Reilly, Institute of Health & Society, Human Nutrition Research Centre, Newcastle University, Newcastle upon Tyne, UK
Mark S Pearce, Institute of Health & Society, Newcastle University, Sir James Spence Institute, Royal Victoria Infirmary, Newcastle upon Tyne, UK
John J Reilly, Physical Activity for Health Group, School of Psychological Sciences and Health, University of Strathclyde, Glasgow, UK
The Gateshead Millennium Study Core Team: Ashley Adamson, Anne Dale, Robert Drewett, Ann Le Couteur, Paul McArdle, Kathryn Parkinson, Mark Pearce, John Reilly, Charlotte Wright

Key words: epidemiologic studies; child; overweight; physical activity; diet

Word count: 3702

For peer review only

For peer review only - http://bmjopen.bmj.com/site/about/guidelines.xhtml

ABSTRACT

Objective

To assess the influence ofrelationships between current physical activity (PA), PA and dietary intake on and body mass index (BMI) in English children.

Design and setting

Longitudinal birth cohort study in northeast England, cross-sectional analysis.

Participants

425 children (41% of the original cohort) aged 6-8 years (49% boys).

Main outcome measures

PA over seven days was measured objectively by accelerometer, three categories of PA were created: 'Active' ≥60min/d moderate-to-vigorous-intensity PA (MVPA); 'moderately active' 30-59min/d MVPA; inactive <30min/d MVPA. Dietary intake over four days was measured using a prospective dietary assessment tool which incorporated elements of both the food diary and food frequency methods. Three diet categories were created: 'healthy'; 'unhealthy' and 'mixed', according to the number of portions of different foods consumed. Adherence to the '5 a day' recommendations for portions of fruit and vegetables was also assessed. Children were classified as 'healthy weight' or 'overweight or obese' (OW/OB) according to International Obesity Taskforce cutpoints for BMI. Associations between weight status and PA/diet categories were analysed using logistic regression.

Results

Few children met the UK recommended guidelines for either MVPA or fruit and vegetable intake, with just 7% meeting the recommended amount of MVPA of 60 mins/d, and 3% meeting the 5-a-day fruit and vegetable recommendation. Higher PA was associated with a lower odds ratio for OW/OB in boys only (0.20 95% CI 0.04-0.88). There was no association detected between dietary intake and OW/OB in either sex.

Conclusions

For peer review only - http://bmjopen.bmj.com/site/about/guidelines.xhtml

Increasing MVPA may help to reduce OW/OB in boys; however, more research is required to examine this relationship in girls. Children are not meeting the UK guidelines for diet and PA, and more needs to be done to improve this situation.

Strengths and limitations of this study

- Direct objectively measured physical activity and observed dietary intake
- Direct measures of body composition
- Population based birth cohort socioeconomically representative of northeast England
- Ethnically homogeneous sample which reduces generalisability
- Cross-sectional so cannot determine direction of causality

For peer review only

INTRODUCTION

Childhood obesity is a major public health challenge both in the UK and around the world, with governments increasingly involving themselves in different strategies to reduce excess weight in the population.[1, 2] Recent findings from the National Child Measurement Programme (2011-2012) regarding the prevalence of childhood overweight and obesity are concerning,[3] especially in northeast England, which is the worst-affected region of England outside of London, where 24.5% of 4-5 year olds and 37.0% of 10-11 year olds are overweight or obese. There remains a limited understanding of the timing and causes of childhood obesity and there is an urgent need for effective preventive strategies.

Current UK health recommendations state that children should accumulate at least 60 minutes of moderate-vigorous intensity physical activity (MVPA) per day, with vigorous intensity activities performed on at least 3 days per week.[4] This recommendation is based on evidence from intervention studies.[5] However, recent evidence suggests that the majority of children fail to meet this target, with 51% of English 4-10 year old boys and 34% of 4-10year old girls achieving the physical activity recommendations as measured by accelerometer, figures which dropped to 7% and 0% among 11-15 year olds.[6] In addition, children should consume a healthy balanced diet which contains a variety of foods, including at least 5 portions of fruit and vegetables per day, plenty of starchy foods (particularly wholegrain varieties), some protein-rich foods and some milk and dairy foods. It is also recommended that foods high in fat (especially saturated fat), salt and sugar should only be consumed in small amounts.[7] Data from the National Diet and Nutrition Survey in 2010 indicated that children aged 4-10 years consume an average of 173g of fruit and vegetables daily, equivalent to 2.2 80g portions (or 2.8 including fruit juice).[8] Data from longitudinal cohorts are beginning to uncover the associations between low levels of physical activity and unfavourable body composition e.g. Janz et al (2009),[9] but the data on the relationship between diet/dietary components and body composition have so far been equivocal, as reviewed by Must et al.[10] This may be due to the contrasting methods of dietary assessment and analysis and the complexity of dietary patterns; given the importance of energy balance in maintenance of a healthy body weight it is vital that measurement of dietary intake continues to be improved. Evidence of the impact of *both* objectively measured physical activity (PA)/sedentary behaviour (SB) *and* food intake on body composition in primary school-age children is lacking, with most studies measuring either physical

For peer review only - http://bmjopen.bmj.com/site/about/guidelines.xhtml

activity *or* food intake. A recent Scottish study assessed the effect of dietary patterns and physical activity on body mass index (BMI),[11] but used a questionnaire that is known to overestimate time spent in physical activity.[12] The authors did not find a consistent association between dietary patterns or time spent in physical activity and BMI group.[11]

The primary aim of the present study was to describe the relationship between overweight and obesity, BMI, PA, and dietary habits in a sample of children that are socioeconomically representative of northeast England.[13]

METHODS

Participants

The Gateshead Millennium Study (GMS) cohort originally consisted of 1029 babies and their families and is described in detail elsewhere.[13] Briefly, all mothers resident in Gateshead who gave birth within pre-specified recruiting weeks between June 1999 and May 2000 were invited to take part, 81% (n=1011) agreed. Participants were mostly from the white ethnic majority (98%). Data collection for the present study involved a variety of lifestyle and anthropometric measures, and took place between October 2006 and December 2007 when the children were aged 6-8 years. The data presented here are cross-sectional data from the 6-8 year is data collection phase of the GMS only. Ethical approval was given by Gateshead and South Tyneside Local National Health Service Research Ethics Committee. Parents provided informed written consent for their child's participation and children gave their written assent.

Measures

Physical activity and sedentary behaviour

PA and SB were objectively measured using Actigraph GT1M accelerometers (Actigraph LLC, Pensacola, Florida, USA) attached to an elastic belt and worn on the right hip as described previously.[14] Children were asked to wear the accelerometer during waking hours for 7 days and to remove them only for water-based activities. Parents were asked to complete a time-log detailing when the accelerometer had been worn.

For peer review only - http://bmjopen.bmj.com/site/about/guidelines.xhtml

The accelerometers collected data in 15 second sampling intervals (epochs); prior to analysis the data were collapsed to 60 second epochs. Data were verified manually using the time-log and accelerometry output as described previously[12] to delete occasional periods of non-wear time.

Three constructs of PA and one of SB were generated: total volume of activity (expressed as mean daily accelerometer counts per minute, cpm); MVPA daily, equivalent to energy expenditure in excess of approximately three times resting energy expenditure (expressed as both minutes per day and % of daily time spent in MVPA), and sedentary behaviour (equivalent to no translocation of the trunk[15] (expressed as % daily time spent in SB)).

The cutpoint of ≥3200 cpm[16] was used to define MVPA; and <1100 cpm[17] was used to define SB. The Actigraph GT1M model has been shown to have a consistent 9% bias relative to model 7164[18] from which the cutpoints were derived, so a +9% correction was applied to the raw accelerometry data before applying cutpoints. Of 606 accelerometers given out, 510 were worn for at least 3 days and for at least 6 hours per day[19] and thus were deemed eligible for analysis in the present study.

Dietary intake

Food intake in the children was assessed over 4 days (2 week days and 2 weekend days) using the 'Food Assessment in Schools Tool' (FAST) food diary.[20] The FAST food diary is a prospective dietary assessment method which incorporates elements of both the food diary and the food frequency methods and was specifically designed to assess the diets of primary school children. Briefly, each day is split into six timeslots, each containing a simple tick list of up to 50 foods commonly eaten by children of this age.[21] Space for additional foods was also provided. Researchers and lay-observers recorded food intake for each child during the school day, and parents and other carers recorded food intake at all other times. No interviews or estimations of portion size were required during data collection, average age- and sex- specific portion sizes for all foods consumed were ascertained using relevant UK National Diet and Nutrition Survey data.[20, 21] This method allowed analysis of food choice at individual level but not of energy intake.

A 'healthy' and 'unhealthy' dietary indicator score was created for each of the four days, and a daily mean calculated. The 'healthy' indicator (HI) score was calculated by summing the number of portions consumed of the following foods: wholemeal bread; non-high sugar wholegrain breakfast cereals; fruit; vegetables; semi-skimmed

For peer review only - http://bmjopen.bmj.com/site/about/guidelines.xhtml

milk as a drink; semi-skimmed milk; non-processed meat and fish products; beans and pulses; water and white bread with added fibre. The 'unhealthy' indicator (UI) score was calculated by summing the number of portions consumed of the following foods: white bread; high-sugar refined breakfast cereals; fried chips; biscuits; confectionery, cakes and sweet puddings; full-fat milk as a drink; full-fat milk; processed meat and fish products; full sugar cordial or squash; full sugar carbonated drinks; chocolate/milkshake powder; crisps and savoury snacks and sugar. A cereal was categorised as 'non-high sugar' if it contained <5g sugar/100g and 'high sugar' if it contained >15g sugar/100g as manufactured. The addition of discretionary sugar was not taken into account when categorising cereals. To assess adherence to dietary guidelines, fruit and vegetable consumption was measured in 80g amounts, fruit juice in 150ml portions. Only one portion of fruit juice may count towards daily fruit and vegetable intake.[7]

Anthropometry

All body measurements were taken in duplicate with the children in light indoor clothing, without socks and shoes. Height was measured to 0.1cm with a Leicester portable height measure (Chasmors, London, UK), waist to 0.1cm at the minimum circumference between the lowest rib and iliac crest, and weight to 0.1kg using a TANITA TBF 300MA body fat analyser (Chasmors, London, UK). BMI was calculated (wt (kg)/ht(m^2)). Categories of underweight, healthy weight, overweight and obesity were calculated according to ~~the~~ age- and sex-specific International Obesity Taskforce (IOTF) criteria.[22, 23]

Statistical analysis

The sample size for the current cross-sectional study was fixed by the size of the cohort, participant attrition and those children providing complete data for PA, dietary intake and anthropometry at this data sweep.

Analysis was performed using SPSS 17 and STATA 10. Shapiro-Wilk tests assessed the normality of distribution of variables. As data were skewed, Mann-Whitney tests were performed to test for differences between sexes and Wilcoxon Rank Tests to assess differences in weekend and weekday PA/SB. Median and interquartile ranges are reported unless otherwise stated.

Logistic regression was used to predict the likelihood of being overweight or obese (OW/OB) at different levels of PA and with different dietary indicator scores. Children were assigned to one of three PA categories: (1) 'active': those who met the ≥60 minutes per day guidelines (this figure was generated by dividing the total amount of

For peer review only - http://bmjopen.bmj.com/site/about/guidelines.xhtml

MVPA for the week by the number of days the accelerometer was worn.); (2) 'moderately active': those who did between 30 and 60 min·d^{-1}; (3) 'inactive': those who did less than 30 min·d^{-1}. Children were also assigned to one of three dietary categories: (1) 'healthy': those children whose HI score was ≥ the median and whose UI score was < the median ; (2) 'unhealthy': those children whose UI score was ≥ the median and whose HI score < the median ; (3) 'mixed': those diets which neither met the criteria for a 'healthy' diet or 'unhealthy' diet. Children were lastly classified by BMI as: (1) not overweight or obese; (2) overweight or obese. Odds ratios (OR) and corresponding 95% confidence intervals (95% CI) are reported. Significance was set at p<0.05. Sex was included in the regression analysis as a potential confounder. Townsend score from birth (a measure of deprivation) was unrelated to any of the variables and thus not included in the final analysis.

RESULTS

Overall, 510 children completed sufficient accelerometry recording, 474 children returned completed FAST food diaries and 597 children had anthropometry measurements. Data from all three domains were provided by 425 children and were used in the analysis. There were no significant differences in food intake, PA or BMI between children providing complete records and those who did not. Children had a mean age of 7.4y (range 6.4-8.4). Characteristics of the sample are displayed in Table 1.

For peer review only - http://bmjopen.bmj.com/site/about/guidelines.xhtml

Table 1 Participant characteristics (median and IQR), by sex

	Combined* (n=425)		Boys (n=211) (49.6%)		Girls (n=214) (50.4%)		p-value
Height (cm)	1.24	1.21, 1.29	1.26	1.22, 1.29	1.24	1.21, 1.28	*0.039*
Weight (kg)	26.4	22.7, 28.7	25.7	23.0, 29.1	25.0	22.5, 28.4	0.264
Waist (cm)	55.7	52.6, 59.0	56.5	53.5, 59.4	54.8	52.1, 58.7	*0.006*
BMI (kg/m^2)	16.2	15.3, 17.8	16.1	15.4, 17.8	16.3	15.2, 17.8	0.878

*n for individual measures varies from 421-425. Mann-Whitney tests performed. BMI, body mass index

Formatted: Font: Italic

Formatted: Font: Italic

For peer review only

Using IOTF cutoffs,[22] 24% of the children were overweight or obese, with no significant difference between girls and boys (Mann-Whitney test, p=0.885). The full distribution of children between weight categories is shown in (Table 2).

Table 2 Distribution of children between weight categories, by sex

	Combined		Boys (49.6%)		Girls (50.4%)	
	n	%	n	%	n	%
Underweight	3	0.7	1	0.5	2	0.9
Healthy weight	320	75.3	160	75.8	160	74.8
Overweight	75	17.6	35	16.6	40	18.7
Obese	27	6.4	15	7.1	12	5.6
Total	425	100	211	100	214	100

There were no significant differences between the sexes in the number of days the activity belt was worn or the total volume of activity (mean cpm) (Table 3).

For peer review only

For peer review only - http://bmjopen.bmj.com/site/about/guidelines.xhtml

Table 3 Comparison of physical activity, sedentary behaviour and dietary intake between sexes (median and IQR unless otherwise specified)

	Combined (n=425)		Boys (n=211)		Girls (n=214)		p-value
Physical activity/sedentary behaviour							
Number of days accelerometer worn	7	6,7	7	6,7	7	6,7	0.380
Hours worn/d	11.3	10.6, 11.9	11.4	10.7, 12.0	11.1	10.4, 11.8	0.077
Mean cpm/d	727	604, 878	740	624, 881	718	591, 868	0.313
MVPA (min/d)	26	18, 38	28	19, 43	25	16, 36	*0.020*
% time in MVPA	3.9	2.6, 5.7	4.2	2.7, 6.3	3.8	2.5, 5.5	*0.043*
% time SB	77.7	73.6, 81.8	76.9	71.9, 80.8	78.4	74.6, 82.7	*0.002*
Achieved 60 min/d MVPA (n, %)*	31	(7.3%)	23	(10.9%)	8	(3.7%)	*0.005*
Dietary intake							
Portions of fruit & vegetables consumed/d	1.7	1.1, 2.5	1.7	1.0, 2.5	1.8	1.2, 2.5	0.211
Portions of fruit & vegetables consumed/d$	2.1	1.4,3.0	2.0	1.3,2.9	2.2	1.4,3.1	0.224
Ate 5 portions of fruit & vegetables/d (n, %)$	13	(3.1%)	8	(3.8%)	5	(2.3%)	0.384
Number of healthy indicator foods/d	5.0	3.8, 6.5	5.0	3.8, 6.5	5.1	3.8, 6.5	0.342
Number of unhealthy indicator foods/d	5.3	4.3, 6.3	5.5	4.3, 6.3	5.3	4.3, 6.3	0.496

MVPA, moderate-vigorous intensity physical activity

*total MVPA for the week/days worn, $including one 150ml portion of juice. Mann-Whitney tests performed.

Formatted Table

Formatted Table

Formatted: Font: Italic

Formatted: Font: Italic

Formatted: Font: Italic

Formatted: Font: Italic

For peer review only

For peer review only - http://bmjopen.bmj.com/site/about/guidelines.xhtml

Levels of activity in general were low, as reported previously; [12] only 31 children (7%) achieved an average of at least 60 min MVPA·d^{-1}, 23 boys and 8 girls. Only two children (0.5%) accumulated at least 60 minutes on every day they wore the accelerometer. The majority of time was spent in sedentary behaviour (median 78% of wear time, equivalent to around 8.6 hours per day). When looking at the differences between weekend and weekday activity, volume of activity (cpm) was similar, and slightly but significantly more MVPA was taken on weekdays ($p<0.0001$) (data not shown). Girls had a greater volume of activity at the weekend but a lower time spent in MVPA, suggesting the majority of their activities are in the sedentary or light intensity categories. Boys spent significantly more time in MVPA on weekdays than girls.

Formatted: Font: Italic

Dietary intake was similar for boys and girls (Table 3). Children consumed a median of 1.7 portions of fruit and vegetables daily (or 2.1 portions when including fruit juice), with just 13/425 children (3.1%) consuming their '5 a day'. On average, children ate 5.0 healthy indicator foods and 5.3 unhealthy indicator foods daily.

Table 4 displays the distribution of overweight/obesity by diet and activity group.

For peer review only - http://bmjopen.bmj.com/site/about/guidelines.xhtml

Table 4. Distribution of OW/OB by physical activity and dietary category (n, %)

	Healthy weight (323, 76.0)				Overweight or obese (102, 24.0)			
	Dietary Pattern				Dietary Pattern			
PA category	Unhealthy	Mixed	Healthy	Total	Unhealthy	Mixed	Healthy	Total
**Boys** (211, 49.6)								
Inactive	27, 16.8	33, 20.5	18, 11.2	78, 48.4	11, 22.0	13, 26.0	14, 28.0	38, 76.0
Moderately active	17, 10.6	28, 17.4	17, 10.6	62, 38.5	2, 4.0	7, 14.0	1, 2.0	10, 20.0
Active	9, 5.6	10, 6.2	2, 1.2	21, 13.0	0, 0.0	1, 2.0	1, 2.0	2, 4.0
Total	53, 32.9	71, 44.1	37, 23.0	161, 100.0	13, 26.0	21, 42.0	16, 32.0	50, 100.0
**Girls** (214, 50.4)								
Inactive	27, 16.7	29, 17.9	40, 24.7	96, 59.3	9, 17.3	18, 34.6	10, 19.2	37, 71.2
Moderately active	17, 10.5	31, 19.1	12, 7.4	60, 37.0	4, 7.7	4, 7.7	5, 9.6	13, 25.0
Active	1, 0.6	3, 1.9	2, 1.2	6, 3.7	0, 0.0	2, 3.8	0, 0.0	2, 3.8
Total	45, 27.8	63, 38.9	54, 33.3	162, 100.0	13, 25.0	24, 46.2	15, 28.8	52, 100.0

For peer review only - http://bmjopen.bmj.com/site/about/guidelines.xhtml

13% of healthy weight boys were in the ‘active’ PA category, compared with just 4% of OW/OB boys. 48% of healthy weight boys were inactive compared with 76% of OW/OB boys. There was less variation for girls, as only 8 girls in total were in the active category. 59% of healthy weight girls were in the inactive category compared with 71% of OW/OB girls.

Compared with the reference group (inactive), children in the moderately active group had a significantly lower risk of being OW/OB (OR=0.44, 0.26-0.74 (Table 5)).

For peer review only

For peer review only - http://bmjopen.bmj.com/site/about/guidelines.xhtml

Table 5 Likelihood of being overweight or obese for different activity levels and dietary patterns (odds ratios and 95% CI)

	Combined (n=425)	p-value	Boys (n=211)	p-value	Girls (n=214)	p-value
Inactive (reference)	1		1		1	
Moderately active	0.44 (0.26-0.74)	*0.002*	0.33 (0.15-0.72)	*0.005*	0.56 (0.28-1.14)	0.112
Active	0.34 (0.12-1.02)	0.054	0.20 (0.04-0.88)	*0.033*	0.86 (0.17-4.48)	0.863
Unhealthy diet (reference)	1		1		1	
Mixed diet	1.27 (0.73-2.19)	0.40	1.21 (0.55-2.62)	0.637	1.32 (0.61-2.86)	0.485
Healthy diet	1.28 (0.71-2.33)	0.41	1.76 (0.76-4.10)	0.188	0.96 (0.42-2.23)	0.927

Formatted: Font: Italic

Formatted: Font: Italic

Formatted: Font: Italic

For peer review only

Results for children in the active group (≥60 min MVPA·d^{-1} (OR=0.34, 95% CI 0.12-1.02) approached significance (p=0.054). Similar results were seen after adjusting for sex. ; PA OR 0.96 p=0.876, diet OR 1.02 p=0.921.

When analysing girls and boys separately, the association between PA and risk of OW/OB remained only for boys. Boys who were moderately active had an OR of 0.33 (0.15-0.72) for risk of OW/OB (*p=0.005*), and boys in the active category had an OR of 0.20 (0.04-0.88) (*p=0.033*), compared with the inactive group. Moderately active girls had an OR of 0.56 (0.28-1.14) (p=0.112) and active girls an OR of 0.86 (0.17-4.48) (p=0.863) for OW/OB compared with inactive girls. Similar results were seen for activity when adjusting for diet group.

Formatted: Font: Italic

Formatted: Font: Italic

There was no influence of dietary pattern on risk of OW/OB: OR 1.27 (0.73-2.19 p=0.40) for the mixed diet group, 1.28 (0.71-2.33) for the healthy group (p=0.41) compared with the reference group (unhealthy diet) (Table 5), with similar results after adjusting for sex.

There was no evidence of an interaction between PA and food intake on risk of OW/OB (p=0.444).

DISCUSSION

Our results provide evidence that children in the north east of England are not meeting the guidelines for physical activity and nutrition that are recommended for good health. Lack of moderate-vigorous intensity physical activity was associated with an increased risk of being overweight and obese in boys. The Llack of an association effect in girls could be due to the low numbers being sufficiently active.

The present study has limitations in terms of the ethnically homogenous sample, which may limit the generalisability of the findings to other samples and settings. There was also a lack of variability in PA, with data skewed towards inactivity, particularly in girls. There are several cut-points in use for classifying MVPA[24] and the value of 3200cpm used in the current study was obtained through comparison with energy expenditure,[16] and similar to those from other studies.[15] Given the on-going discussion regarding the choice of cut-point there may be no suitable cut-point for all ages of childhood. As the data are cross-sectional, it is not possible to determine the direction of causality, which will be addressed by future follow up. However, the strengths of this study include the relatively large sample size, the

For peer review only - http://bmjopen.bmj.com/site/about/guidelines.xhtml

focused age of the children, and the use of objective measures of PA (accelerometry) combined with dietary observations is unique.

Few other studies have used objective methods to describe levels of habitual physical activity and sedentary behaviour in 6-8 year olds in combination with dietary intake measures. Recent UK studies in 3-5 year olds[14] and 11-12 year olds[25] which have used the same objective methods of PA measurement have found similarly low levels of physical activity with adherence to the 60 minutes/day recommendation of MVPA being achieved in <5% of their samples. In contrast, UK studies which have used subjective methods (questionnaires) have generally reported much higher levels of MVPA, for example the Health Survey for England, which leads to over-estimates of PA.[12] Low levels of physical activity were present in our sample well before puberty, the period traditionally regarded as being associated with low levels of physical activity. In keeping with other studies[9], [26], we found that boys were more active than girls, and the lack of association of PA on risk of OW/OB in the girls compared with the boys suggests further work is required into the mechanisms by which increasing PA and/or decreasing SB reduces risk of OW/OB in children. Few studies have looked at the relationship between the '60min' guidelines and body composition. Martinez-Gomez et al (2010)[27] reported that 60 min·d^{-1} MVPA could discriminate between normal weight and OW/OB adolescents (mean age 14.7 years), however they used a lower cut-point for MVPA of 2000cpm, compared with the 3200cpm used in the present study. This resulted in average MVPA·d^{-1} of 66 minutes for boys and 50 minutes for girls.[27] Recent evidence using 3200cpm as the MVPA cutpoint from 10 year olds in France[28] found that only 10% of the 'normal weight' children reached 60 min·d^{-1}, but 44% did at least 30 min·d^{-1}. In addition, although only 5% of OW/OB children did at least 60min·d^{-1} MVPA, 34% reached 30 min·d^{-1}.[28] Our results show that this lower recommendation could still have benefits in terms of body composition, particularly for boys.

Whilst it is generally accepted that physical activity decreases with age,[29] pinpointing the age when the decrease starts is important for targeted interventions. Our results suggest that even at age 7 years physical activity levels are far lower than recommended. The great proportion of time spent sedentary by both sexes is concerning - in both sexes almost 7 hours per day were spent with no translocation of the trunk.[15] Both sexes took part in more MVPA on weekdays than weekends, highlighting both the importance the school environment plays in maintaining activity levels, and that more needs to be done to increase activity at the weekend. The cross-sectional nature of the present study means we cannot say whether the

For peer review only

children have already started to reduce their activity. However, data we have published from the cohort at a 2 year follow-up show a decrease in MVPA from age 7 to 9 years, and the decrease in MVPA associated with an increase in BMI-z score in boys.[30, 31]

The low consumption of fruit and vegetables, and the relatively high consumption of an unhealthy diet in this sample are both cause for concern, despite the lack of association between the 'healthy' or 'unhealthy' categories and risk of OW/OB. The lack of association between dietary variables and risk of OW/OB in this study, as in others, may be due to the lack of individual portion sizes which prohibited the assessment of energy intake. The dietary assessment tool used in this study allowed direct observation of intake but applied standard portion sizes from a previous National Diet and Nutrition Survey which used a weighed intake method.[21] The amount for a 'portion' of fruits and vegetables was set at 80g, which is considered suitable for children aged over 11 years.[32] A lower amount may be more suitable for children under 11 years, but as this has not yet been identified,[32] 80g was used in this analysis. It is possible that overweight children are eating larger portions of healthy foods, or the parents are aware of their child's overweight and have reduced food intake accordingly. There may also be an element of mis-reporting or optimistic bias if the parent believes their child is overweight and eats either to excess or 'unhealthily'. Foods in the current study were assigned to the 'healthy' or 'unhealthy' group though a combination of current national guidelines for consumption and restriction[7] according to nutritional content. Since these analyses were performed, Frémeaux and colleagues[33] have published a longitudinal factor analysis of English children's food intake from age 5 to 13 years. They reported the emergence of two main diet types that matched almost identically with ours.[33] Recent evidence from Scottish 5-11 year olds did not find an association between dietary patterns and BMI or physical activity,[11] and observed an inverse association between 'screen time' and healthier eating patterns (diet measure with food frequency questionnaire, FFQ, and physical activity measured by questionnaire, PAQ). The ALSPAC cohort in the UK were assessed for associations between PA and diet at age 10-11 years, and found only weak relationships between the two[34] (3x1-day food diary at 10y, accelerometry at 11y). A Greek case-control study found a protective effect of PA[35] on risk of OW/OB, and an increased risk with increased time spent watching TV, and consumption of sugar-sweetened beverages (FFQ/PAQ), an observation also reported in Spanish children (FFQ/PAQ).[36] Others [37] found an effect of sedentary behaviour on risk of OW/OB, but again no effect of diet composition (questionnaire).

For peer review only - http://bmjopen.bmj.com/site/about/guidelines.xhtml

A varied food pattern plus a physically active lifestyle was inversely related to OW in French children (7-day food diary/PAQ).[38] Similarly, reduced physical activity, but not excessive energy intake, was associated with increased risk of OB in Turkish children (3-day food diary/PAQ).[39] However, these studies included a wide age range of children, and physical activity and sedentary behaviour were measured objectively only in the ALSPAC cohort[34], so our observations strengthen the evidence base for the protective effects of PA on risk of OW/OB.

In conclusion, children in northeast England have extremely low levels of habitual MVPA, high levels of sedentary behaviour and consume a diet low in fruits and vegetables but high in foods associated with an unhealthy diet. Increased PA was associated with reduced risk of OW/OB in boys. Further work is required to uncover the associations of PA/diet and OW/OB in girls and the longitudinal associations between PA/diet and OW/OB in boys and girls. These observations give cause for concern in relation to their current and future risk of cardiovascular diseases, diabetes, and obesity. Our observations suggest that UK government targets in relation to levels of physical activity and nutrition among children are not being met.

ACKNOWLEDGEMENTS

The Gateshead Millennium Study is supported by a grant from the National Prevention Research Initiative (incorporating funding from British Heart Foundation; Cancer Research UK; Department of Health; Diabetes UK; Economic and Social Research Council; Food Standards Agency; Medical Research Council; Research and Development Office for the Northern Ireland Health and Social Services; Chief Scientist Office, Scottish Government Health Directorates; Welsh Assembly Government and World Cancer Research Fund). The cohort was first established with funding from the Henry Smith Charity and Sport Aiding Research in Kids (SPARKS) and followed up with grants from Gateshead NHS Trust R&D, Northern and Yorkshire NHS R&D, and Northumberland, Tyne and Wear NHS Trust. We acknowledge the support of an External Reference Group in conducting the study. We appreciate the support of Gateshead Health NHS Foundation Trust, Gateshead Education Authority and local schools. We warmly thank the research team for their effort. Thanks are especially due to the Gateshead Millennium Study families and children for their participation in the study.

For peer review only - http://bmjopen.bmj.com/site/about/guidelines.xhtml

FUNDING

This work was supported by the National Prevention Research Initiative (NPRI) grant number GO 501306.

COMPETING INTERESTS

None declared.

For peer review only

For peer review only - http://bmjopen.bmj.com/site/about/guidelines.xhtml

REFERENCES

1. Department of Health. Healthy lives, healthy people: A call to action on obesity in England. London, 2011.

2. www.letsmove.gov. Let's Move! , 2010,pWebsite for Michelle Obama's campaign to reduce childhood obesity.

3. The Health and Social Care Information Centre. National Child Measurement Programme: England, 2011/2012 school year. 2012.

4. Chief Medical Officers of England Scotland Wales and Northern Ireland. Start active, stay active: a report on physical activity from the four home countries' Chief Medical Officers. Department of Health: London, 2011.

5. Strong WB, Malina RM, Blimkie CJR*, et al.* Evidence based physical activity for school-age youth. *J Pediatr* 2005;**146:**732-737.

6. Health Survey for England 2008. Volume 1: Physical activity and fitness. In: Craig R, Mindell J, Hirani V (eds), 2009.

7. NHS Choices. Healthy Eating. 2013.

8. Department of Health. National Diet and Nutrition Survey. Headline results from Years 1 and 2 (combined) of the Rolling Programme (2008/2009 - 2009/2010). In: Bates B, Lennox A, Bates C, Swan G (eds). HMSO: London, 2011.

For peer review only - http://bmjopen.bmj.com/site/about/guidelines.xhtml

9. Janz KF, Kwon S, Letuchy EM*, et al.* Sustained Effect of Early Physical Activity on Body Fat Mass in Older Children. *Am J Prev Med* 2009;**37:**35-40.

10. Must A, Barish EE, Bandini LG. Modifiable risk factors in relation to changes in BMI and fatness: what have we learned from prospective studies of school-aged children[quest]. *Int J Obes* 2009;**33:**705-715.

11. Craig LCA, McNeill G, Macdiarmid JI*, et al.* Dietary patterns of school-age children in Scotland: association with socio-economic indicators, physical activity and obesity. *Br J Nutr* 2009;**103:**319-334.

12. Basterfield L, Adamson AJ, Parkinson KN*, et al.* Surveillance of physical activity in the UK is flawed: Validation of the Health Survey for England Physical Activity Questionnaire. *Arch Dis Child* 2008;**93:**1054-1058.

13. Parkinson KN, Pearce MS, Dale A*, et al.* Cohort Profile: The Gateshead Millennium Study. *Int J Epidemiol* 2010;**40:**308-317.

14. Reilly JJ, Jackson DM, Montgomery C*, et al.* Total energy expenditure and physical activity in young Scottish children: mixed longitudinal study. *Lancet* 2004;**363:**211-212.

15. Reilly JJ, Penpraze V, Hislop J*, et al.* Objective measurement of physical activity and sedentary behaviour: Review with new data. *Arch Dis Child* 2008;**93:**614-619.

16. Puyau MR, Adolph AL, Vohra FA*, et al.* Validation and calibration of physical activity monitors in children. *Obes Res* 2002;**10:**150-157.

For peer review only

For peer review only - http://bmjopen.bmj.com/site/about/guidelines.xhtml

17. Reilly JJ, Coyle J, Kelly L, *et al.* An objective method for measurement of sedentary behavior in 3- to 4-year olds. *Obes Res* 2003;**11:**1155-1158.

18. Corder K, Brage S, Ramachandran A, *et al.* Comparison of two Actigraph models for assessing free-living physical activity in Indian adolescents. *J Sports Sci* 2007;**25:**1607-1611.

19. Basterfield L, Adamson AJ, Pearce MS, *et al.* Stability of habitual physical activity and sedentary behavior monitoring by accelerometry in 6-8 year olds. *J Phys Act Health* 2011;**8:**543-547.

20. Adamson AJ, Griffiths JM, Carlin LE, *et al.* FAST: Food Assessment in Schools Tool. *Proc Nutr Soc* 2003;**62:**84A.

21. Gregory JR, Lowe S, Bates CJ, *et al.* National Diet and Nutrition Survey: Young People Aged 4 to 18 Years. Volume 1: Report of the Diet and Nutrition Survey. The Stationary Office: London, 2000.

22. Cole TJ, Bellizzi MC, Flegal KM, *et al.* Establishing a standard definition for child overweight and obesity worldwide: international survey. *Br Med J* 2000;**320:**1240-1243.

23. Cole TJ, Flegal KM, Nicholls D, *et al.* Body mass index cut offs to define thinness in children and adolescents: international survey. *Br Med J* 2007;**335:**194-197.

For peer review only - http://bmjopen.bmj.com/site/about/guidelines.xhtml

24. Trost SG, Loprinzi PD, Moore R, *et al.* Comparison of Accelerometer Cut Points for Predicting Activity Intensity in Youth. *Med Sci Sports Exerc* 2011;**43:**1360-1368.

25. Riddoch CJ, Mattocks C, Deere K, *et al.* Objective measurement of levels and patterns of physical activity. *Arch Dis Child* 2007;**92:**963-969.

26. Riddoch CJ, Andersen LB, Wedderkopp N, *et al.* Physical activity levels and patterns of 9-and 15-yr-old European children. *Med Sci Sports Exerc* 2004;**36:**86-92.

27. Martinez-Gomez D, Ruiz JR, Ortega FB, *et al.* Recommended Levels of Physical Activity to Avoid an Excess of Body Fat in European Adolescents The HELENA Study. *Am J Prev Med* 2010;**39:**203-211.

28. Apete GK, Zitouni D, Hubert H, *et al.* Compliance of children in northern France with physical activity recommendations. *Perspect Public Health* 2012;**132:**81-88.

29. Jago R, Wedderkopp N, Kristensen PL, *et al.* Six-Year Change in Youth Physical Activity and Effect on Fasting Insulin and HOMA-IR. *Am J Prev Med* 2008;**35:**554-560.

30. Basterfield L, Adamson AJ, Frary JK, *et al.* Longitudinal study of physical activity and sedentary behavior in children. *Pediatrics* 2011;**127:**E24-E30.

31. Basterfield L, Pearce MS, Adamson AJ, *et al.* Physical activity, sedentary behavior, and adiposity in English children. *Am J Prev Med* 2012;**42:**445-451.

For peer review only - http://bmjopen.bmj.com/site/about/guidelines.xhtml

32. Ruxton CHS, Derbyshire E. Diet adequacy in UK schoolchildren. *Nutrition & Food Science* 2011;**41:**20-33.

33. Fremeaux AE, Hosking J, Metcalf BS*, et al.* Consistency of children's dietary choices: annual repeat measures from 5 to 13 years (EarlyBird 49). *Br J Nutr* 2011;**106:**725-731.

34. Jago R, Ness AR, Emmett P*, et al.* Obesogenic diet and physical activity: independent or associated behaviours in adolescents? *Public Health Nutr* 2010;**13:**673-681.

35. Papandreou D, Malindretos P, Rousso I. Risk factors for childhood obesity in a Greek paediatric population. *Public Health Nutr* 2010;**First View:**1-5.

36. Ochoa MC, Moreno-Aliaga MJ, Martínez-González MA*, et al.* Predictor factors for childhood obesity in a Spanish case-control study. *Nutrition* 2007;**23:**379-384.

37. Lagiou A, Parava M. Correlates of childhood obesity in Athens, Greece. *Public Health Nutr* 2008;**11:**940-945.

38. Lioret S, Touvier M, Lafay L*, et al.* Dietary and Physical Activity Patterns in French Children Are Related to Overweight and Socioeconomic Status. *J Nutr* 2008;**138:**101-107.

39. Garipagaoglu M, Budak N, Süt N*, et al.* Obesity risk factors in Turkish children. *J Pediatr Nurs* 2009;**24:**332-337.

For peer review only

AUTHOR CONTRIBUTIONS

AJA and KNP were responsible for the study conception and design, manuscript drafting and revision. AJA is the study guarantor. JJR and MSP were responsible for the study conception and design, analysis and interpretation of data, manuscript drafting and revision. LB and ARJ were responsible for data acquisition, analysis and interpretation of data; manuscript drafting and revision. All authors have had full access to all of the data in the study and take responsibility for the integrity of the data and the accuracy of the data analysis

For peer review only - http://bmjopen.bmj.com/site/about/guidelines.xhtml

STROBE Statement—Checklist of items that should be included in reports of ***cross-sectional studies***

	Item No	Whether included	Page or table in submitted manuscript	Article and page of uploaded supplementary material	Recommendation
**Title and abstract**	1	YES	Page 1: Title		(*a*) Indicate the study's design with a commonly used term in the title or the abstract
		YES	Page 2-3: Abstract		(*b*) Provide in the abstract an informative and balanced summary of what was done and what was found
**Introduction**					
Background/rationale	2	YES	Page 4: Introduction		Explain the scientific background and rationale for the investigation being reported
Objectives	3	YES	Page 5: Introduction	Gateshead Millennium Study protocol Upload 1 Page 3-4	State specific objectives, including any prespecified hypotheses
**Methods**					
Study design	4	YES	Page 5, 6 & 7: Methods Participants section Measures section	Gateshead Millennium Study protocol Upload 1 Page 4-6	Present key elements of study design early in the paper
Setting	5	YES	Page 5: Methods Participants section	Gateshead Millennium Study protocol Upload 1 Page 4-6	Describe the setting, locations, and relevant dates, including periods of recruitment, exposure, follow-up, and data collection
Participants	6	YES	Page 5: Methods Participants section	Gateshead Millennium Study protocol Upload 1 Page 4 Parkinson et al (2011) Int J Epi – Cohort Profile.	(*a*) Give the eligibility criteria, and the sources and methods of selection of participants

For peer review only - http://bmjopen.bmj.com/site/about/guidelines.xhtml

For peer review only

Variables	7	YES	Page 5-7: Methods Measures section	Parkinson et al (2011) Int J Epi – Cohort Profile.	Clearly define all outcomes, exposures, predictors, potential confounders, and effect modifiers. Give diagnostic criteria, if applicable
Data sources/ measurement	8*	YES	Page 5-7: Methods	Gateshead Millennium Study protocol Upload 1 Page 4	For each variable of interest, give sources of data and details of methods of assessment (measurement). Describe comparability of assessment methods if there is more than one group
Bias	9	YES		Parkinson et al (2011) Int J Epi – Cohort Profile	Describe any efforts to address potential sources of bias
Study size	10	YES	Page 7: Methods Statistical analysis section		Explain how the study size was arrived at
Quantitative variables	11	YES	Page 7: Methods Statistical analysis section		Explain how quantitative variables were handled in the analyses. If applicable, describe which groupings were chosen and why
Statistical methods	12	YES	Page 7: Methods Statistical analysis section		(*a*) Describe all statistical methods, including those used to control for confounding
		YES	Page 7: Methods Statistical analysis section		(*b*) Describe any methods used to examine subgroups and interactions
		YES	Minimal; page 8: Results section first paragraph		(*c*) Explain how missing data were addressed
		N/A			(*d*) If applicable, describe analytical methods taking account of sampling strategy
		N/A			(*e*) Describe any sensitivity analyses
**Results**					
Participants	13*	YES	Page 8: Results	Parkinson et al (2011) Int J Epi – Cohort Profile.	(a) Report numbers of individuals at each stage of study—eg numbers potentially eligible, examined for eligibility, confirmed eligible, included in the study, completing follow-up, and analysed
		NO		Parkinson et al (2011) Int J Epi – Cohort Profile.	(b) Give reasons for non-participation at each stage
		N/A			(c) Consider use of a flow diagram

For peer review only - http://bmjopen.bmj.com/site/about/guidelines.xhtml

For peer review only

Descriptive data	14*	YES	Table 1	(a) Give characteristics of study participants (eg demographic, clinical, social) and information on exposures and potential confounders
		YES	Page 8: Results	(b) Indicate number of participants with missing data for each variable of interest
Outcome data	15*	YES	Tables 2, 3, 4.	Report numbers of outcome events or summary measures
Main results	16	YES	Page 16: Results<br>Table 5	(*a*) Give unadjusted estimates and, if applicable, confounder-adjusted estimates and their precision (eg, 95% confidence interval). Make clear which confounders were adjusted for and why they were included
		YES	Page 7	(*b*) Report category boundaries when continuous variables were categorized
		Not relevant		(*c*) If relevant, consider translating estimates of relative risk into absolute risk for a meaningful time period
Other analyses	17	YES	Page 16<br>Results	Report other analyses done—eg analyses of subgroups and interactions, and sensitivity analyses
**Discussion**				
Key results	18	YES	Page 16: Discussion<br>Paragraph 1	Summarise key results with reference to study objectives
Limitations	19	YES	Pages 16: Discussion<br>Paragraph 2	Discuss limitations of the study, taking into account sources of potential bias or imprecision. Discuss both direction and magnitude of any potential bias
Interpretation	20	YES	Pages 16 - 19: Discussion	Give a cautious overall interpretation of results considering objectives, limitations, multiplicity of analyses, results from similar studies, and other relevant evidence
Generalisability	21	YES	Pages 16 – 19: Discussion	Discuss the generalisability (external validity) of the study results
**Other information**				
Funding	22	YES	Page 20	Give the source of funding and the role of the funders for the present study and, if applicable, for the original study on which the present article is based

For peer review only - http://bmjopen.bmj.com/site/about/guidelines.xhtml

*Give information separately for exposed and unexposed groups.

Note: An Explanation and Elaboration article discusses each checklist item and gives methodological background and published examples of transparent reporting. The STROBE checklist is best used in conjunction with this article (freely available on the Web sites of PLoS Medicine at http://www.plosmedicine.org/, Annals of Internal Medicine at http://www.annals.org/, and Epidemiology at http://www.epidem.com/). Information on the STROBE Initiative is available at www.strobe-statement.org.

For peer review only

For peer review only - http://bmjopen.bmj.com/site/about/guidelines.xhtml
